# Effects of vaccination and non-pharmaceutical interventions and their lag times on the COVID-19 pandemic: Comparison of eight countries

**DOI:** 10.1371/journal.pntd.0010101

**Published:** 2022-01-13

**Authors:** Hao Li, Luqi Wang, Mengxi Zhang, Yihan Lu, Weibing Wang

**Affiliations:** 1 School of Public Health, Fudan University, Shanghai, China; 2 Key Laboratory of Public Health Safety of Ministry of Education, Fudan University, Shanghai, China; Minia University, EGYPT

## Abstract

Many countries implemented measures to control the COVID-19 pandemic, but the effects of these measures have varied greatly. We evaluated the effects of different policies, the prevalence of dominant variants (e.g., Delta), and vaccination on the characteristics of the COVID-19 pandemic in eight countries. We quantified the lag times of different non-pharmaceutical interventions (NPIs) and vaccination using a distributed lag non-linear model (DLNM). We also tested whether these lag times were reasonable by analyzing changes in daily cases and the effective reproductive number (R_t_)over time. Our results indicated that the response to vaccination in countries with continuous vaccination programs lagged by at least 40 days, and the lag time for a response to NPIs was at least 14 days. A rebound was most likely to occur during the 40 days after the first vaccine dose. We also found that the combination of school closure, workplace closure, restrictions on mass gatherings, and stay-at-home requirements were successful in containing the pandemic. Our results thus demonstrated that vaccination was effective, although some regions were adversely affected by new variants and low vaccination coverage. Importantly, relaxation of NPIs soon after implementation of a vaccination program may lead to a rebound.

## Author summary

In response to contain the COVID-19 pandemic, countries worldwide have implemented multiple non-pharmaceutical interventions (NPIs) that restrict movement, such as school closure and stay-at-home requirements, and introduced large nationwide COVID-19 vaccination campaigns. However, there were variations in population compliance and vaccine coverage worldwide. The virus is continuously spreading and new genetic variants are emerging. In this context, we chose eight countries as being representative of the worldwide ranges of population density, variant spread, vaccination, population compliance to countermeasures, and effectiveness of implemented measures. Our study identified the most common effective countermeasures and estimated the lag times for a response to NPIs and for the onset of a vaccination effect. We found a rebound was most likely to occur during the 40 days after the first vaccine dose and both NPIs and vaccine coverage are indispensable in the current context of Delta variant high prevalence. We hope that the results of this study could provide some guidance for controlling the pandemic.

## Introduction

The coronavirus disease 2019 (COVID-19), caused by SARS-CoV-2, has spread throughout the world since its initial identification in December 2019, and has had devastating effects in many countries. As of August 31, 2021, there were over 215 million confirmed cases worldwide, with the Americas (39%) and Europe (30%) accounting for most cases [1]. Because SARS-CoV-2 is highly contagious and effective treatments are not available, multiple public health measures have been implemented in an effort to control this pandemic. It has been reported that more than 6000 non-pharmaceutical interventions (NPIs, the most important countermeasures before vaccinations became available) were implemented across 261 countries and territories [2].

Large nationwide COVID-19 vaccination campaigns began worldwide at the beginning of 2021. However, there were variations in the efficacy of the different vaccines and their availability in different countries. As of August 31, 2021, Singapore (75.21%), United Kingdom (62.91%), Israel (62.45%), and United States (51.77%) have high shares of the population fully vaccinated against COVID-19, whereas this proportion was relatively low in Australia (27.80%) and India (10.61%) [3]. An observational study in Israel reported that the adjusted estimates of BNT162b2 vaccine effectiveness at 7 days or longer after the second dose was95.3% [4]. A study in England reported that the BNT162b2 vaccine had an effectiveness of 85% at 7 days after the second dose [5]. A study in Chile reported that the estimated vaccine effectiveness of inactived vaccine (CoronaVac) was 65.9% [6].

SARS-CoV-2 continuously spread, and multiple genetic variants have been reported [7]. Most mutations occurred in viral spike (S) protein, which enables the virus to enter into a host cell by binding to angiotensin converting enzyme 2 (ACE2) receptor [8]. The identified mutants began with the D641G variants and now include the well-known variants of concern (VOCs), Alpha (B.1.1.7; from the United Kingdom), Beta (B.1.351; from South Africa), Gamma (P.1; from Brazil), and Delta (B.1.617.2; from India) [9]; the variants differ in their transmissibility and immune evasiveness. It has been reported that the current vaccines are still effective (as of September 1, 2021), preventing over 90% of severe disease even when facing the highly contagious Delta variant. However, breakthrough and reinfected cases are increasing as expected, and the ability of the vaccines to prevent the spread of the virus remains unclear [10].

Previous studies have documented the remarkable effectiveness of NPIs in controlling the COVID-19 pandemic, but these studies mainly focused on the early pandemic without considering vaccination and/or the rapid evolution and increasing number of VOCs [11]. Therefore, we herein evaluated the effectiveness and potential lag times of NPIs during the whole course of the pandemic, within the context of Delta variant spreading and vaccination, in eight representative countries from January 21, 2020 to August 31, 2021. Our general goal was to provide evidence that can be used to facilitate the effective containment of COVID-19 and identify the most appropriate public health countermeasures.

## Materials and methods

### Data sources

We chose eight countries as being representative of the worldwide ranges of population density, variant spread, vaccination, population compliance to countermeasures, and effectiveness of implemented measures. The included countries were Australia, Israel, India, Japan, Singapore, South Korea, the United Kingdoms (UK), and the United States (US). Basic information on the COVID-19 epidemics in these eight countries was obtained from publicly available data sources [3,12] (Table 1 and S1). The start dates and coverage of COVID-19 vaccinations were from official reports in each country [13]. The proportion of Delta variant was obtained from public database (https://github.com/owid/covid-19-data/tree/master/public/data) [14].

**Table 1:** Basic characteristics of the selected countries.

Characteristic	Australia	Japan	Israel	United Kingdom	United States	South Korea	Singapore	India
Population in millions	25.5	126.5	8.65	67.9	331.0	51.27	5.85	1380.0
Population density per km^2^	3.202	347.778	402.606	272.898	35.608	527.967	7915.731	450.419
Age 70 years or older (%)	10.129	18.493	7.359	12.527	9.732	8.622	7.049	3.414
GDP per person (USD)	44,648.71	39,002.22	33,132.32	39,753.24	54,225.45	35,938.37	85,535.38	6,426.674
Extreme poverty (%)	0.5	-	0.5	0.2	1.2	0.2	-	21.2
Hospital beds per thousand	3.84	13.05	2.99	2.54	2.77	12.27	2.4	0.53
Life expectancy (years)	83.44	84.63	82.97	81.32	78.86	83.03	83.62	69.66
Human development index	0.944	0.919	0.919	0.922	0.926	0.916	0.938	0.645
GHS	75.5	59.8	47.3	77.9	83.5	70.2	58.7	46.5
Start date of vaccination (dd/mm/yy)	22/2/21	17/2/21	19/12/20	3/1/21	20/12/20	25/2/21	11/1/21	15/1/21
Vaccine coverage (%)*	27.80	46.42	62.45	62.91	51.77	30.70	75.21	10.61
Initial vaccine	Pfizer/AstraZeneca	Pfizer	Pfizer	Pfizer	Pfizer	Pfizer/AstraZeneca	Pfizer/Sinovac	AstraZeneca/Covaxin

GDP, Gross Domestic Product; GHS, Global health security; *2021/08/31

The policy responses were derived from the open-access Oxford COVID-19 Government Response Tracker (OxCGRT) [15]. The Oxford team collects information on common policy responses, scores the stringency of these measures, and establishes a Stringency Index. Policy and response categories were coded as follows: school closure (C1); workplace closure (C2); restrictions on public events (C3); restrictions on mass gatherings (C4); closure of public transport (C5); stay-at-home requirements (C6); restrictions on internal movement (C7); and international travel controls (C8). Each policy had three to five levels, and the stringency was rated as 0 (no policy), 1 (recommendation), and 2 or above (requirement). Explaining of each policy of levels is provided in S2 Table. Effective reproductive number (R_t_) for each country was taken from the study by Arroyo-Marioli et al. [16]. Standard Kalman-filtering was used in the study [16] to smooth out the noise in the observed growth rate of total cases. R_t_ is a key epidemiological parameter, and was used to determine whether an epidemic was increasing (R_t_>1), decreasing (R_t_<1), or remaining steady after changes in policies, population immunity, and other factors that can affect transmission at specific times and locations.

### Statistical analysis

First, during the exploratory phase, policy responses, R_t_ values and R package DLNM were performed in R(v4.0.3) [17] to construct a distributed lag non-linear model (DLNM) for each country to calculate relative risk (RR) of the eight different policies (C1–C8) and to estimate the lag time (duration from the initiation of an action to an observable effect) for each policy. DLNM is a common approach for testing lag effects in epidemiological and environmental studies; it is helpful in guiding policy-making and evaluating policy effectiveness. The lag time for the onset of a vaccination effect was estimated to be 40 days, which is approximately equal to 14 days (the duration between two vaccine doses) plus 28 days (from taking the second dose to the sufficient antibodies in the body). In the part of evaluating policy effects, the data used for the DLNM analysis were drawn from the time the first patient was diagnosed to 40 days after the first dose of the vaccine for avoiding policy effects confounded by vaccination. Based on estimations from the DLNMs, the effectiveness of different policies in each country and their associated lag times were determined.

Second, during the validation phase, the four policies found in our exploratory phase to play a major role were analyzed. These protective policies demonstrated efficacy in most countries at 14 days after introduction, and thus a 14-day lag time was used for each one. The vaccination lag time was set at 40 days. The effects of changes during each phase, fluctuations in the number of daily new cases per million people and R_t_ were plotted, and the major VOC were marked. The generated images offer clear illustrations of how NPIs and vaccination impacted on the COVID-19 pandemic. In addition, the appropriateness of each lag times was assessed.

## Results

### Exploratory analysis of effective policies and lag time

The results of our DLNM analysis indicated that school closure (C1), workplace closure (C2), restrictions on mass gatherings (C4), and stay-at-home requirements (C6) had the desired protective effects, as indicated by declines in relative risk (RR) in S1–S8 Figs. According to our estimates, most of the studied policies had an effect after 14 days. School closure (C1) had the greatest effect in Australia, when the level was upgraded from “no measures” to “closing all levels schools” (relative risk [RR]: 0.667; 95% CI: 0.585, 0.761; lag time: 14 days). Workplace closure (C2) had the greatest effect in Israel, when the level was upgraded from “no measures” to “required closing (or work from home) for all-but-essential workplaces” (RR: 0.073; 95% CI: 0.042, 0.134; lag time: 14 days). Restrictions on mass gatherings (C4) had the strongest effect in Australia, when the level was upgraded from “no measures” to “restrictions on gatherings of 10 people or fewer” (RR: 0.238; 95% CI: 0.179, 0.316; lag time: 14 days). Stay at home requirements (C6) had the strongest effect in the US, when the level was upgraded from “no measures” to a requirement for “not leaving the house with exceptions for daily exercise, grocery shopping, and essential trips” (RR: 0.600; 95% CI: 0.542, 0.633; lag time: 14 days). Table 2 indicated that the RR of C1 and C2 measures or C1, C2, C4, and C6 measures was below 1.0 in eight studied countries. Detailed RR values and 95% confidence intervals with lag time and response stringencies were presented in S3 Table.

**Table 2:** The RR values and 95% confidence intervals of C1 and C2 or C1, C2, C4, and C6 with lag time in eight studies countries.

Locations	C12			C1246				
	Lag days	RR	95% CI	Lag days	RR	95% CI	Mean Lag days	Rank
Australia	7	0.945	(0.906 to 0.987)	21	0.976	(0.968 to 0.984)	14.0	5
India	19	0.994	(0.992 to 0.996)	16	0.994	(0.992 to 0.996)	17.5	1
Israel	16	0.583	(0.530 to 0.641)	13	0.981	(0.970 to 0.992)	14.5	4
Japan	12	0.976	(0.965 to 0.988)	7	0.911	(0.848 to 0.978)	9.5	7
Singapore	15	0.867	(0.848 to 0.886)	15	0.867	(0.848 to 0.886)	15	2
South Korea	10	0.770	(0.675 to 0.878)	11	0.916	(0.873 to 0.961)	10.5	6
the United Kingdom	19	0.916	(0.884 to 0.950)	11	0.973	(0.957 to 0.989)	15.0	2
the United States	7	0.955	(0.932 to 0.979)	7	0.955	(0.932 to 0.979)	7.0	8

### Validation and identification of influencing factors

**Fig 1:**
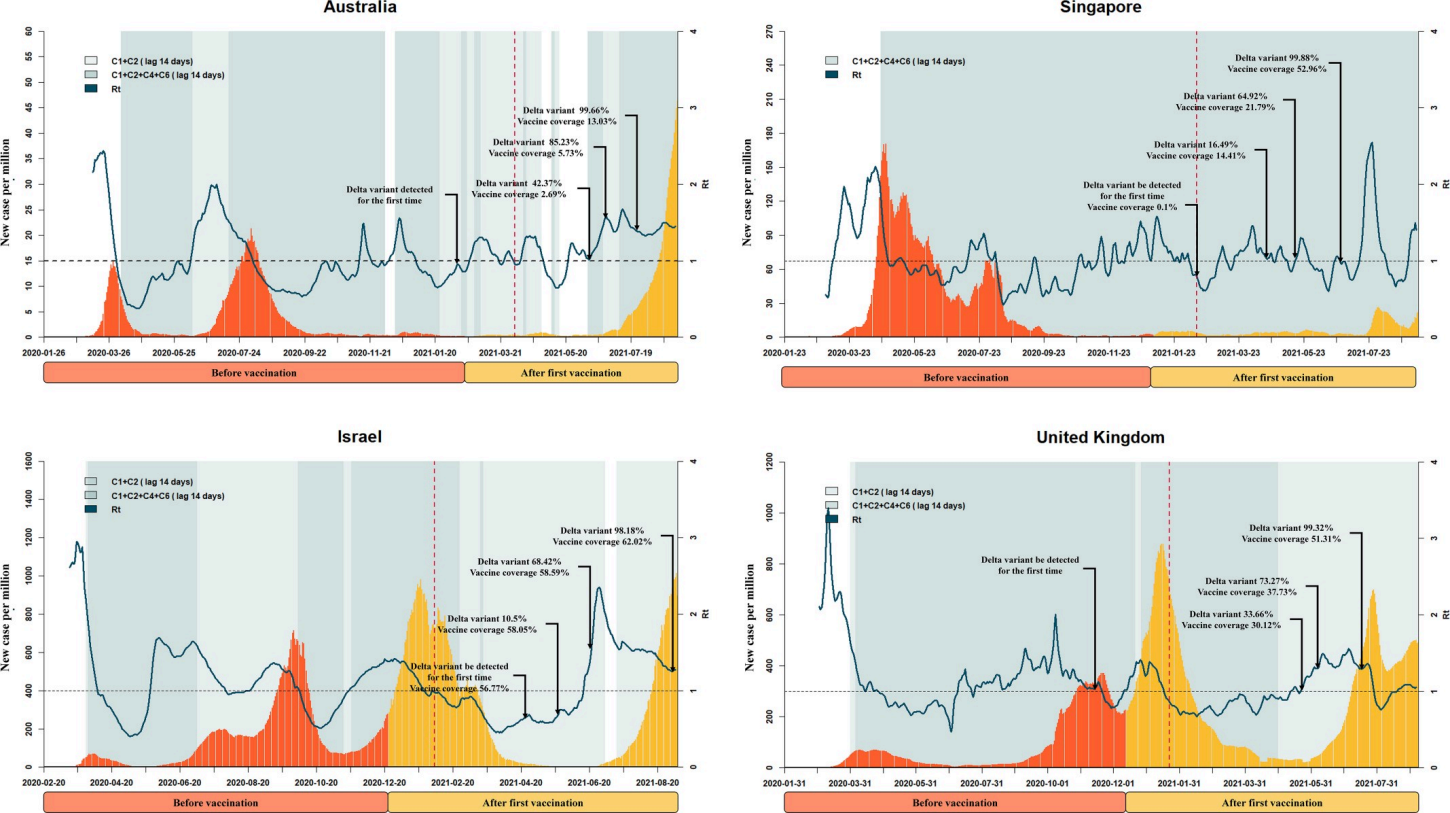
Key events during the progression of the pandemic in the Australia, Singapore, Singapore and United Kingdom. The x axis represents time. The y axis represents daily new confirmed cases. The fluctuation line represents the effective reproductive number (R_t_). The shading represents the implementation of a public health measure plus 14 days (lag time). The light-to-dark color shift indicates a gradual increase of stringency. The dotted vertical line represents the announcement of vaccination plus 40 days (lag time). Some key proportions of Delta variant and vaccine coverage are marked using dark arrows.

**Fig 2:**
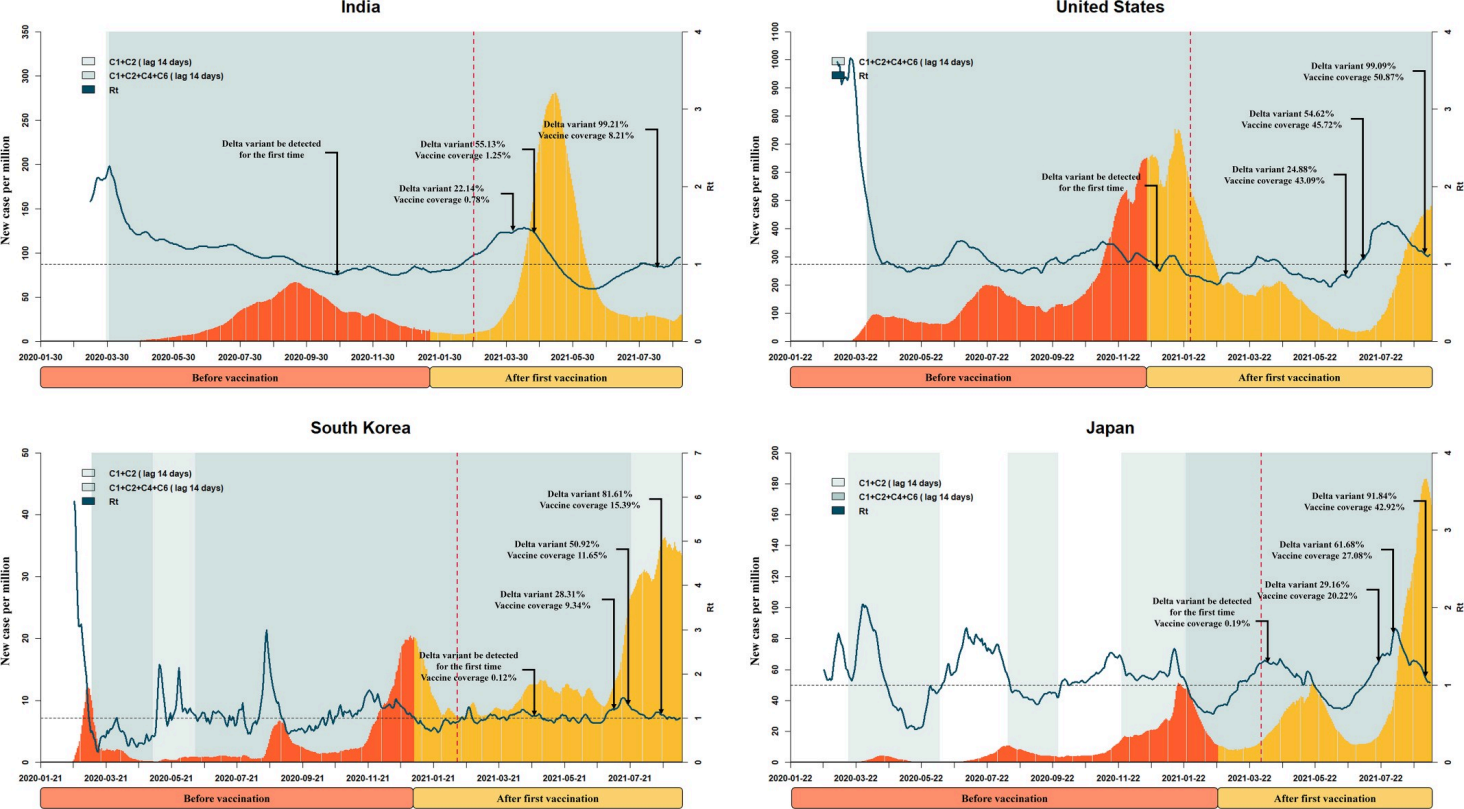
Key events during the progression of the pandemic in India, United States, South Korea and Japan. The x axis represents time. The y axis represents daily new confirmed cases. The fluctuation line represents the effective reproductive number (R_t_). The shading represents the implementation of a public health measure plus 14 days (lag time). The light-to-dark color shift indicates an increase of stringency. The dotted vertical line represents the announcement of vaccination plus 40 days (lag time). Some key proportions of Delta variant and vaccine coverage are marked using dark arrows.

In Figs 1 and 2, during the early phase of the pandemic, especially when variants had not yet been detected, all eight countries experienced decreasing R_t_ values due to the implementation of C1 and C2 measures or C1, C2, C4, and C6 measures after a 14-day lag time for the onset of policy effect (beginning of the shading). This is consistent with the findings from exploratory phase. After the first dose of vaccine, the number of new cases continued to increase in Australia, Japan, Israel, Singapore, the UK, and the US, and the R_t_ value of each country fluctuated. During this time, the pandemic was very likely to rebound. After a 40-day lag time for the onset of vaccination effect (dotted vertical lines), countries whose vaccination programs had more than 30% coverage by June 14, 2021 (Israel, Singapore, the UK, and the US) exhibited decreases in the numbers of daily new cases during the period when the Delta variant had not yet begun to dominate. However, nearly all of the studied countries reported Delta variant proportions as high as 90% by July 2021, and nearly all experienced a more severe third or fourth wave of disease as the Delta variant became dominant. That said, the daily new cases in Australia, Singapore, and South Korea remained under 50 per million as of August 31, 2021.

**Fig 3:**
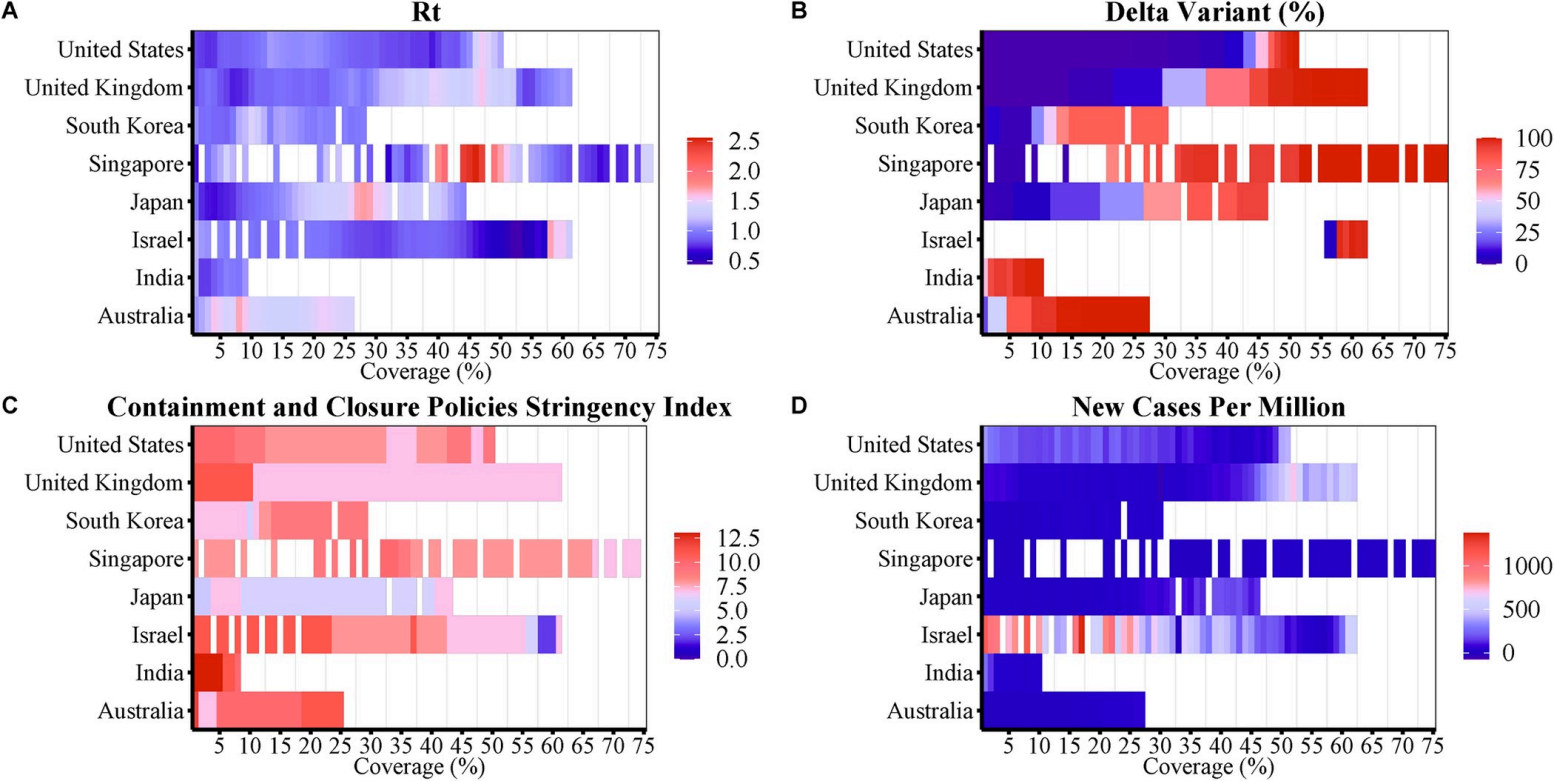
Association of vaccine coverage with R_t_ (A), new cases per million (B), containment and closure policies stringency index (C) and Delta variant proportion (D) in eight different countries.

Fig 3A shows that the R_t_ fluctuated 1.0 in eight studied countries before vaccination, however, it increased slightly as the vaccine coverage increased in the UK Singapore, Japan, and Australia, which had high proportions of Delta variant (Fig 3B), though Singapore and Japan maintained fairly consistent response stringencies with their precious times (Fig 3C). Australia had an increasing stringency index but a low vaccine coverage. The UK had a high vaccine coverage but a decreasing stringency index in the later phrase of pandemic. In Israel, the daily number of new confirmed cases fell rapidly as the vaccine coverage increased (Fig 3D) despite decreasing stringency indexes in the absence of Delta variant.

## Discussion

In this study, we estimated and verified the value of the lag time for containment and closure policies and vaccination in eight representative countries, and found four policies that play key role in controlling COVID-19 with considering the prevalence of variants and current vaccine coverage. We found that a rebound was most likely to occur during the 40 day period immediately after the onset of a vaccination program and that in the current context of Delta variant prevalence, both public health measures and vaccine coverage are indispensable. Since April 2020, the epidemic has been largely under controlled in China, with typically fewer than 0.01 daily new confirmed cases [18], but spread throughout the world [19]. For each of the eight countries analyzed here, the R_t_ declined sharply after the adoption of prevention and control policies and then fluctuated around 1.0 (depending on policies and country) throughout the whole study period. Early containment was therefore especially important in these eight countries, as previously documented for China [20,21]. Our results revealed that school closure (C1), workplace closure (C2), restrictions on mass gatherings (C4), and stay-at-home requirements (C6) played key roles, which is consistent with the findings of other studies [22–24]. We found that Japan was highly sensitive to containment policies [25] and that the pandemic is well-controlled (<50 daily new cases per million) before August 2021, even though this country adopted fewer policies at less stringent levels. We found that the other countries implemented the C1, C2, C4, and C6 policies for most of the study period, and this led to rapid declines of daily new cases in most of these countries. The exceptions were the US and India, which were insensitive to policy changes due to their general populations having relatively low awareness of the importance of prevention and control measures, people did not adopt public health protective measures in time [25]. In India, moreover, the fragile health system ran out of oxygen, drugs, and beds, which exacerbated the second wave of disease in April 2021 [26].

The pandemics in Australia and Singapore were under control beginning in September 2020, but resurgence has recently been seen in these two countries (August 2021), with Delta variant dominating the prevalence. Indeed, Delta variant is leading the third or fourth waves of the global COVID-19 pandemic. All eight countries reported a high prevalence of Delta variant, and the number of new cases increased with the proportion of Delta variant [27,28]. Delta variant was reported as early as October 2020 in India, but it did not play a significant role in driving the epidemic at that time. When Delta variant began to dominate after April 2021, the number of new cases increased sharply, even though the vaccine has exerted efficacy. This is because Delta has two mutations in the spike protein (E484Q and L452R) that allow it to better evade the human immune system [29]. Delta can cause breakthrough infections among fully vaccinated people and spread to more people with the help of its high transmissibility and transmission period [30,31]. As of August 31, 2021, the daily new cases in Australia, Singapore, and South Korea were relatively low (<50 per million). Australia has a low population density and rising stringency index, which appears to be coping with a low vaccine coverage and high Delta variant proportion. Singapore has the highest vaccine coverage and joint implementation of the four effective policies, which appears to be coping with a high population density and high proportion of Delta variant to yield the smallest increase in daily new cases. In South Korea, the Delta variant proportion is relatively low (81%) compared to that in the other countries (91%). The pandemic in Japan was well controlled prior to August 2021, whereupon a high prevalence of Delta variant (>90%) and the withdrawal of 1.63 million doses of Moderna vaccine due to contamination concerns contributed to a more severe third wave of disease despite increases in the stringency index of countermeasures [32].

The use of vaccines in combination with NPIs appears to have the greatest effect in controlling the pandemic [33]. Patel et al. [34] also found that premature lifting of NPIs while vaccines are distributed may result in substantial increases in infections. Although the first vaccine dose provides protection, the analyses presented in Figs 2 and 3 indicated that new daily cases continued to increase afterwards, especially in Israel, the UK, the US, and Japan. This is an important reminder that an epidemic is most likely to rebound soon after the initiation of a vaccination program. Public health officials should therefore pay particular attention to this period and implement appropriate policies. We also noticed that the time when Delta variants were first detected in the UK and US around the time when vaccinations began. The simultaneous presence of new genetic variants and relaxation of NPIs after the initial vaccine doses might have contributed to the increases in new cases [35,36].

Vaccine coverage played an important role beginning 40 days after the first vaccination. At this time, the number of daily new cases declined in the US, the UK, and Israel (which had high coverage). However, low vaccine coverage and the prevalence of Delta variants jointly contributed to increases in the numbers of daily new cases in South Korea and Japan. The greater population density and more frequent gatherings in India might have facilitated the transmission of COVID-19 [37]. Although Israel and India have similar population densities and median age (Table 1), they have very large differences in per capita income and availability of hospital beds. This points to the importance of timely access to medical services and vaccines. Singapore also has a high population density, but its epidemic was mostly controlled, possibly because of the rapid response and cooperation from residents [38]. Both Japan and Israel used the Pfizer vaccine, but the vaccination rate in Israel was 10 times higher than in Japan; this may be why daily new cases in Japan did not decline soon after the onset of vaccination [39]. Israel, Singapore, the US, and the UK all had high vaccination coverages compared to the average level worldwide [3], and daily new cases in these countries began to decline at 40 days after the first vaccination. Thus, although NPIs are essential, vaccine coverage is indispensable [40].

There were some limitations to this study. First, we were unable to identify the most effective interventions for controlling the COVID-19 epidemic because R_t_ is affected by the source of infection, the route of transmission, and population vulnerability [41]. Second, we used data for entire countries, and there were certainly regional differences in policies and epidemiological parameters within individual countries. In particular, the stringency of policy implementation was affected by local factors and was difficult to estimate. We also did not consider some factors that could have affected disease transmission, such as the proportions of different genetic variants, the effects of different vaccines and doses, and other NPIs.

## Conclusion

We herein determined that the lag time for vaccination was 40 days, during which a rebound of the epidemic is most likely. In contrast, the lag time for NPIs was 14 days, and the combined use of C1, C2, C4, and C6 policies had significant effects in containing the COVID-19 pandemic. Despite the long lag time of vaccination, the presence of variants, and the failure to achieve herd immunity, however, our analyses indicate that vaccination is highly effective. Together, our results show that NPIs remain necessary to prevent continued spread of COVID-19 given the current vaccine coverage.

## Supporting information

S1 Fig The effectiveness of the school-closing policy (C1). (DOCX)

S2 Fig The effectiveness of the workplace-closing policy (C2). (DOCX)

S3 Fig The effectiveness of the restrictions on public events policy (C3). (DOCX)

S4 Fig The effectiveness of the restrictions on gatherings policy (C4). (DOCX)

S5 Fig The effectiveness of the closing public transport policy (C5). (DOCX)

S6 Fig The effectiveness of the staying-at-home requirements policy (C6). (DOCX)

S7 Fig The effectiveness of the restrictions on internal movement policy (C7). (DOCX)

S8 Fig The effectiveness of the international travel controls policy (C8). (DOCX)

S9 Fig Association of vaccine coverage with R_t_, new cases per million, containment and closure policies stringency index and Delta variant proportion in Australia. (DOCX)

S10 Fig Association of vaccine coverage with R_t_, new cases per million, containment and closure policies stringency index and Delta variant proportion in Israel. (DOCX)

S11 Fig Association of vaccine coverage with R_t_, new cases per million, containment and closure policies stringency index and Delta variant proportion in Singapore. (DOCX)

S12 Fig Association of vaccine coverage with R_t_, new cases per million, containment and closure policies stringency index and Delta variant proportion in the United Kingdom. (DOCX)

S13 Fig Association of vaccine coverage with R_t_, new cases per million, containment and closure policies stringency index and Delta variant proportion in India. (DOCX)

S14 Fig Association of vaccine coverage with R_t_, new cases per million, containment and closure policies stringency index and Delta variant proportion in the United States. (DOCX)

S15 Fig Association of vaccine coverage with R_t_, new cases per million, containment and closure policies stringency index and Delta variant proportion in South Korea. (DOCX)

S16 Fig Association of vaccine coverage with R_t_, new cases per million, containment and closure policies stringency index and Delta variant proportion in Japan. (DOCX)

S17 Fig Flow chart. (DOCX)

S1 Table Basic and policy data. (XLSX)

S2 Table Levels of each policy. (XLSX)

S3 Table Detailed RR values. (XLSX)

## References

[1] https://www.who.int/publications/m/item/weekly-epidemiological-update-on-covid-19—31-august-2021

[2] Suryanarayanan P, Tsou CH, Poddar A, Mahajan D, Dandala B, Madan P, et al. AI-assisted tracking of worldwide non-pharmaceutical interventions for COVID-19. Sci Data. 2021;8(1):94. Epub 2021/03/27. doi: 10.1038/s41597-021-00878-y ; PubMed Central PMCID: PMC7994304. 33767205 PMC7994304

[3] Mathieu E, Ritchie H, Ortiz-Ospina E, Roser M, Hasell J, Appel C, et al. A global database of COVID-19 vaccinations. Nat Hum Behav. 2021;5(7):947–53. Epub 2021/05/12. doi: 10.1038/s41562-021-01122-8 . 33972767

[4] Haas EJ, Angulo FJ, McLaughlin JM, Anis E, Singer SR, Khan F, et al. Impact and effectiveness of mRNA BNT162b2 vaccine against SARS-CoV-2 infections and COVID-19 cases, hospitalisations, and deaths following a nationwide vaccination campaign in Israel: an observational study using national surveillance data. Lancet. 2021;397(10287):1819–29. Epub 2021/05/09. doi: 10.1016/S0140-6736(21)00947-8 ; PubMed Central PMCID: PMC8099315. 33964222 PMC8099315

[5] Hall VJ, Foulkes S, Saei A, Andrews N, Oguti B, Charlett A, et al. COVID-19 vaccine coverage in health-care workers in England and effectiveness of BNT162b2 mRNA vaccine against infection (SIREN): a prospective, multicentre, cohort study. Lancet. 2021;397(10286):1725–35. Epub 2021/04/27. doi: 10.1016/S0140-6736(21)00790-X ; PubMed Central PMCID: PMC8064668. 33901423 PMC8064668

[6] Jara A, Undurraga EA, González C, Paredes F, Fontecilla T, Jara G, et al. Effectiveness of an Inactivated SARS-CoV-2 Vaccine in Chile. N Engl J Med. 2021;385(10):875–84. Epub 2021/07/08. doi: 10.1056/NEJMoa2107715 ; PubMed Central PMCID: PMC8279092. 34233097 PMC8279092

[7] Jacob JJ, Vasudevan K, Pragasam AK, Gunasekaran K, Veeraraghavan B, Mutreja A. Evolutionary Tracking of SARS-CoV-2 Genetic Variants Highlights an Intricate Balance of Stabilizing and Destabilizing Mutations. mBio. 2021;12(4):e0118821. Epub 2021/07/21. doi: 10.1128/mBio.01188-21 ; PubMed Central PMCID: PMC8406184. 34281387 PMC8406184

[8] Veeramachaneni GK, Thunuguntla V, Bobbillapati J, Bondili JS. Structural and simulation analysis of hotspot residues interactions of SARS-CoV 2 with human ACE2 receptor. J Biomol Struct Dyn. 2021;39(11):4015–25. Epub 2020/05/26. doi: 10.1080/07391102.2020.1773318 ; PubMed Central PMCID: PMC7284149. 32448098 PMC7284149

[9] World Health Organization, SARS-CoV-2 Variants of Concern and Variants of Interest. World Health Organization; 2021. Available from: https://www.who.int/en/activities/tracking-SARS-CoV-2-variants.

[10] The Lancet Respiratory M. Realising the potential of SARS-CoV-2 vaccines-a long shot? Lancet Respir Med. 2021;9(2):117. Epub 2021/01/24. doi: 10.1016/S2213-2600(21)00045-X ; PubMed Central PMCID: PMC7817463. 33484632 PMC7817463

[11] Brauner JM, Mindermann S, Sharma M, Johnston D, Salvatier J, Gavenčiak T, et al. Inferring the effectiveness of government interventions against COVID-19. Science. 2021;371(6531). Epub 2020/12/17. doi: 10.1126/science.abd9338 ; PubMed Central PMCID: PMC7877495. 33323424 PMC7877495

[12] https://www.ghsindex.org/wp-content/uploads/2019/10/2019-Global-Health-Security-Index.pdf

[13] Hasell J, Mathieu E, Beltekian D, Macdonald B, Giattino C, Ortiz-Ospina E, et al. A cross-country database of COVID-19 testing. Sci Data. 2020;7(1):345. Epub 2020/10/10. doi: 10.1038/s41597-020-00688-8 ; PubMed Central PMCID: PMC7545176. 33033256 PMC7545176

[14] Elbe S., and Buckland-Merrett G. (2017) Data, disease and diplomacy: GISAID’s innovative contribution to global health. Global Challenges, 1:33–46. doi: 10.1002/gch2.1018 . 31565258 PMC6607375

[15] Hale T, Angrist N, Goldszmidt R, Kira B, Petherick A, Phillips T, et al. A global panel database of pandemic policies (Oxford COVID-19 Government Response Tracker). Nat Hum Behav. 2021;5(4):529–38. Epub 2021/03/10. doi: 10.1038/s41562-021-01079-8 . 33686204

[16] Arroyo-Marioli F, Bullano F, Kucinskas S, Rondón-Moreno C. Tracking R of COVID-19: A new real-time estimation using the Kalman filter. PLoS ONE. 2021;16(1):e0244474. Epub 2021/01/14. doi: 10.1371/journal.pone.0244474 ; PubMed Central PMCID: PMC7806155. 33439880 PMC7806155

[17] Gasparrini A. Distributed Lag Linear and Non-Linear Models in R: The Package dlnm. J Stat Softw. 2011;43(8):1–20. Epub 2011/10/18. ; PubMed Central PMCID: PMC3191524 22003319 PMC3191524

[18] Xue E, Li J, Li T, Shang W. China’s education response to COVID-19: A perspective of policy analysis. Educational Philosophy and Theory. 2021;53(9):881–93.

[19] Li H, Liu Z, Ge J. Scientific research progress of COVID-19/SARS-CoV-2 in the first five months. J Cell Mol Med. 2020;24(12):6558–70. Epub 2020/04/23. doi: 10.1111/jcmm.15364 ; PubMed Central PMCID: PMC7264656. 32320516 PMC7264656

[20] Kasilingam D, Sathiya Prabhakaran SP, Rajendran DK, Rajagopal V, Santhosh Kumar T, Soundararaj A. Exploring the growth of COVID-19 cases using exponential modelling across 42 countries and predicting signs of early containment using machine learning. Transbound Emerg Dis. 2021;68(3):1001–18. Epub 2020/08/05. doi: 10.1111/tbed.13764 ; PubMed Central PMCID: PMC7436699. 32749759 PMC7436699

[21] Middelburg RA, Rosendaal FR. COVID-19: How to make between-country comparisons. Int J Infect Dis. 2020;96:477–81. Epub 2020/05/30. doi: 10.1016/j.ijid.2020.05.066 ; PubMed Central PMCID: PMC7250090. 32470605 PMC7250090

[22] Wong MC, Huang J, Teoh J, Wong SH. Evaluation on different non-pharmaceutical interventions during COVID-19 pandemic: An analysis of 139 countries. J Infect. 2020;81(3):e70–e1. Epub 2020/06/25. doi: 10.1016/j.jinf.2020.06.044 ; PubMed Central PMCID: PMC7306102. 32579981 PMC7306102

[23] Fuller JA, Hakim A, Victory KR, Date K, Lynch M, Dahl B, et al. Mitigation Policies and COVID-19-Associated Mortality—37 European Countries, January 23-June 30, 2020. MMWR Morb Mortal Wkly Rep. 2021;70(2):58–62. Epub 2021/01/15. doi: 10.15585/mmwr.mm7002e4 ; PubMed Central PMCID: PMC7808713 Journal Editors form for disclosure of potential conflicts of interest. No potential conflicts of interest were disclosed. 33443494 PMC7808713

[24] Woskie LR, Hennessy J, Espinosa V, Tsai TC, Vispute S, Jacobson BH, et al. Early social distancing policies in Europe, changes in mobility & COVID-19 case trajectories: Insights from Spring 2020. PLoS ONE. 2021;16(6):e0253071. Epub 2021/07/01. doi: 10.1371/journal.pone.0253071 ; PubMed Central PMCID: PMC8244916 policies on sharing data and materials. 34191818 PMC8244916

[25] Hou Z, Du F, Zhou X, Jiang H, Martin S, Larson H, et al. Cross-Country Comparison of Public Awareness, Rumors, and Behavioral Responses to the COVID-19 Epidemic: Infodemiology Study. J Med Internet Res. 2020;22(8):e21143. Epub 2020/07/24. doi: 10.2196/21143 ; PubMed Central PMCID: PMC7402643. 32701460 PMC7402643

[26] Wise J. Covid-19: Countries rally to support India through "storm that has shaken the nation". BMJ. 2021;373:n1086. Epub 2021/04/28. doi: 10.1136/bmj.n1086 . 33903127

[27] Tan JB, Cook MJ, Logan P, Rozanova L, Wilder-Smith A. Singapore’s Pandemic Preparedness: An Overview of the First Wave of COVID-19. Int J Environ Res Public Health. 2020;18(1). Epub 2021/01/06. doi: 10.3390/ijerph18010252 ; PubMed Central PMCID: PMC7795346. 33396275 PMC7795346

[28] Hossain MK, Hassanzadeganroudsari M, Apostolopoulos V. The emergence of new strains of SARS-CoV-2. What does it mean for COVID-19 vaccines? Expert Rev Vaccines. 2021:1–4. Epub 2021/04/27. doi: 10.1080/14760584.2021.1915140 ; PubMed Central PMCID: PMC8074646. 33896316 PMC8074646

[29] Alexandar S, Ravisankar M, Kumar RS, Jakkan K. A Comprehensive Review on Covid-19 Delta variant. International Journal of Pharmacology and Clinical Research (IJPCR). 2021;5:83–5.

[30] Chia PY, Ong SWX, Chiew CJ, Ang LW, Chavatte J-M, Mak T-M, et al. Virological and serological kinetics of SARS-CoV-2 Delta variant vaccine-breakthrough infections: a multi-center cohort study. medRxiv. 2021. doi: 10.1016/j.cmi.2021.11.010 34826623 PMC8608661

[31] Nguyen KV. Problems associated with antiviral drugs and vaccines development for COVID-19: approach to intervention using expression vectors via GPI anchor. Nucleosides Nucleotides Nucleic Acids. 2021;40(6):665–706. Epub 2021/05/14. doi: 10.1080/15257770.2021.1914851 ; PubMed Central PMCID: PMC8127170. 33982646 PMC8127170

[32] https://rafu.com/2021/08/japan-suspends-1-63m-doses-of-moderna-over-contamination/

[33] Rowan NJ, Moral RA. Disposable face masks and reusable face coverings as non-pharmaceutical interventions (NPIs) to prevent transmission of SARS-CoV-2 variants that cause coronavirus disease (COVID-19): Role of new sustainable NPI design innovations and predictive mathematical modelling. Sci Total Environ. 2021;772:145530. Epub 2021/02/14. doi: 10.1016/j.scitotenv.2021.145530 ; PubMed Central PMCID: PMC7848491. 33581526 PMC7848491

[34] Patel MD, Rosenstrom E, Ivy JS, Mayorga ME, Keskinocak P, Boyce RM, et al. The Joint Impact of COVID-19 Vaccination and Non-Pharmaceutical Interventions on Infections, Hospitalizations, and Mortality: An Agent-Based Simulation. medRxiv. 2021. Epub 2021/01/15. doi: 10.1101/2020.12.30.20248888 ; PubMed Central PMCID: PMC7805476. 33442712 PMC7805476

[35] Bertuzzo E, Mari L, Pasetto D, Miccoli S, Casagrandi R, Gatto M, et al. The geography of COVID-19 spread in Italy and implications for the relaxation of confinement measures. Nat Commun. 2020;11(1):4264. Epub 2020/08/28. doi: 10.1038/s41467-020-18050-2 ; PubMed Central PMCID: PMC7449964. 32848152 PMC7449964

[36] Yang J, Marziano V, Deng X, Guzzetta G, Zhang J, Trentini F, et al. Despite vaccination, China needs non-pharmaceutical interventions to prevent widespread outbreaks of COVID-19 in 2021. Nat Hum Behav. 2021. Epub 2021/06/24. doi: 10.1038/s41562-021-01155-z . 34158650 PMC8373613

[37] Dubey AP, Hazarika RD, Abitbol V, Kolhapure S, Agrawal S. Mass gatherings: a review of the scope for meningococcal vaccination in the Indian context. Hum Vaccin Immunother. 2021;17(7):2216–24. Epub 2021/02/20. doi: 10.1080/21645515.2020.1871572 ; PubMed Central PMCID: PMC8189129. 33605845 PMC8189129

[38] Abdullah WJ, Kim S. Singapore’s responses to the COVID-19 outbreak: A critical assessment. The American Review of Public Administration. 2020;50(6–7):770–6.

[39] Awadasseid A, Wu Y, Tanaka Y, Zhang W. Effective drugs used to combat SARS-CoV-2 infection and the current status of vaccines. Biomed Pharmacother. 2021;137:111330. Epub 2021/02/08. doi: 10.1016/j.biopha.2021.111330 ; PubMed Central PMCID: PMC7843108. 33550043 PMC7843108

[40] Human rights and fair access to COVID-19 vaccines: the International AIDS Society-Lancet Commission on Health and Human Rights. Lancet. 2021;397(10284):1524–7. Epub 2021/03/28. doi: 10.1016/S0140-6736(21)00708-X ; PubMed Central PMCID: PMC7990478. 33773117 PMC7990478

[41] Guth S, Hanley KA, Althouse BM, Boots M. Ecological processes underlying the emergence of novel enzootic cycles: Arboviruses in the neotropics as a case study. PLoS Negl Trop Dis. 2020;14(8):e0008338. Epub 2020/08/14. doi: 10.1371/journal.pntd.0008338 ; PubMed Central PMCID: PMC7425862. 32790670 PMC7425862

