# Supplementary material for: Effects of vaccination and non-pharmaceutical interventions and their lag times on the COVID-19 pandemic: Comparison of eight countries
Source: PLoS Negl Trop Dis. 2022 Jan 13;16(1):e0010101. doi: 10.1371/journal.pntd.0010101 (PMC8757886; doi:10.1371/journal.pntd.0010101)
Supplement: S1 Fig — (DOCX) [file pntd.0010101.s001.docx]

S1 Fig shows that adopting the school-closing policy (C1) was protective for the majority of countries (RR<1), dangerous for the United States and South Korea (RR>1), and ineffective for the United Kingdom (RR 1.00~1.02).


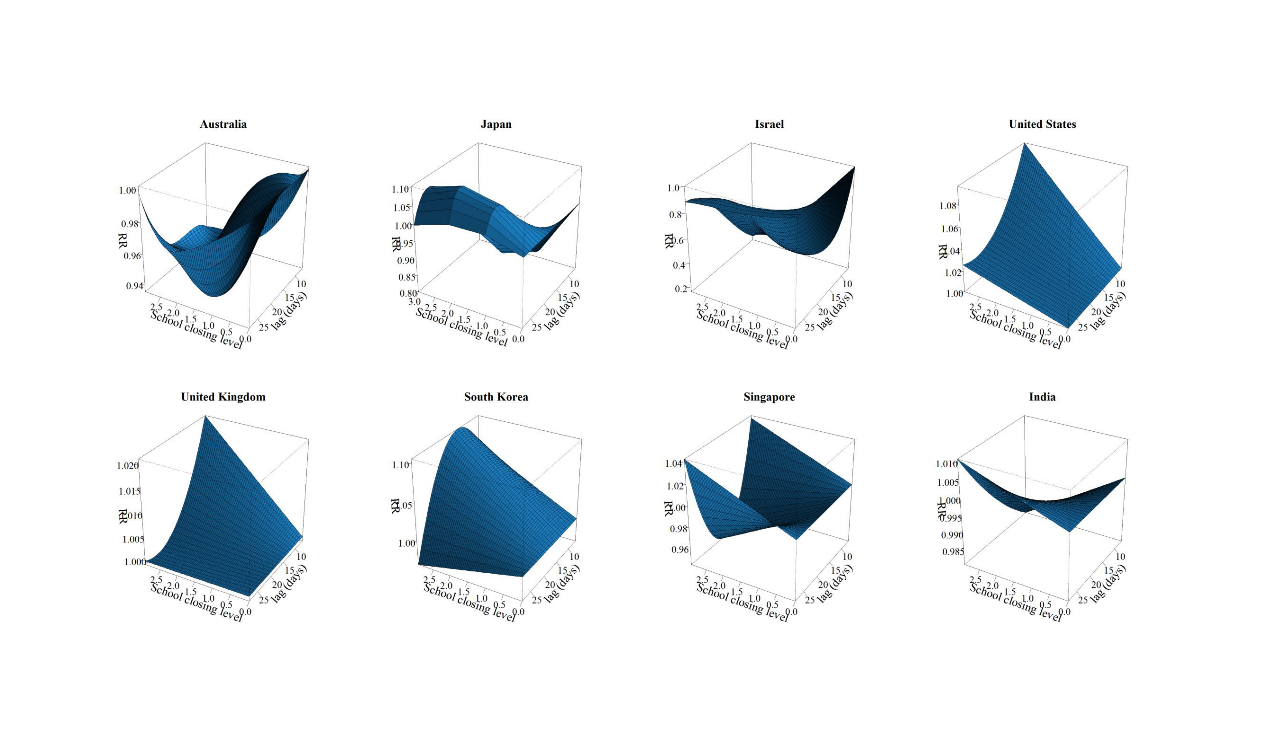
S1 Fig. The effectiveness of the school-closing policy (C1).
