# Supplementary material for: Effects of vaccination and non-pharmaceutical interventions and their lag times on the COVID-19 pandemic: Comparison of eight countries
Source: PLoS Negl Trop Dis. 2022 Jan 13;16(1):e0010101. doi: 10.1371/journal.pntd.0010101 (PMC8757886; doi:10.1371/journal.pntd.0010101)
Supplement: S2 Fig — (DOCX) [file pntd.0010101.s002.docx]

S2 Fig shows that adopting the workplace-closing policy (C2) was protective for the majority of countries (RR<1), but dangerous for Japan and the United Kingdom (RR>1).


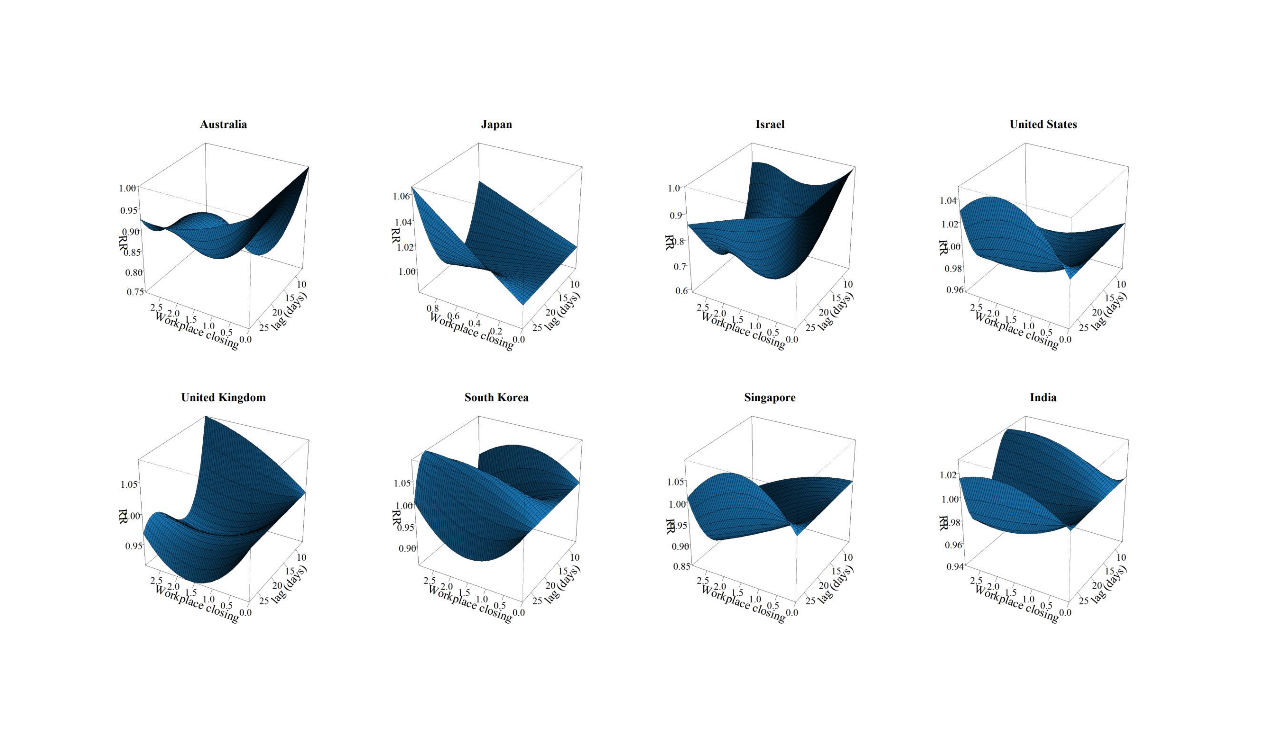
S2 Fig. The effectiveness of the workplace-closing policy (C2).
