# Supplementary material for: Effects of vaccination and non-pharmaceutical interventions and their lag times on the COVID-19 pandemic: Comparison of eight countries
Source: PLoS Negl Trop Dis. 2022 Jan 13;16(1):e0010101. doi: 10.1371/journal.pntd.0010101 (PMC8757886; doi:10.1371/journal.pntd.0010101)
Supplement: S3 Fig — (DOCX) [file pntd.0010101.s003.docx]

S3 Fig shows that the restrictions on public events policy (C3) was protective for the majority of countries (RR<1), but dangerous for the United States and India (RR>1). As C3 was not adopted in Singapore, it could not be evaluated for this country.


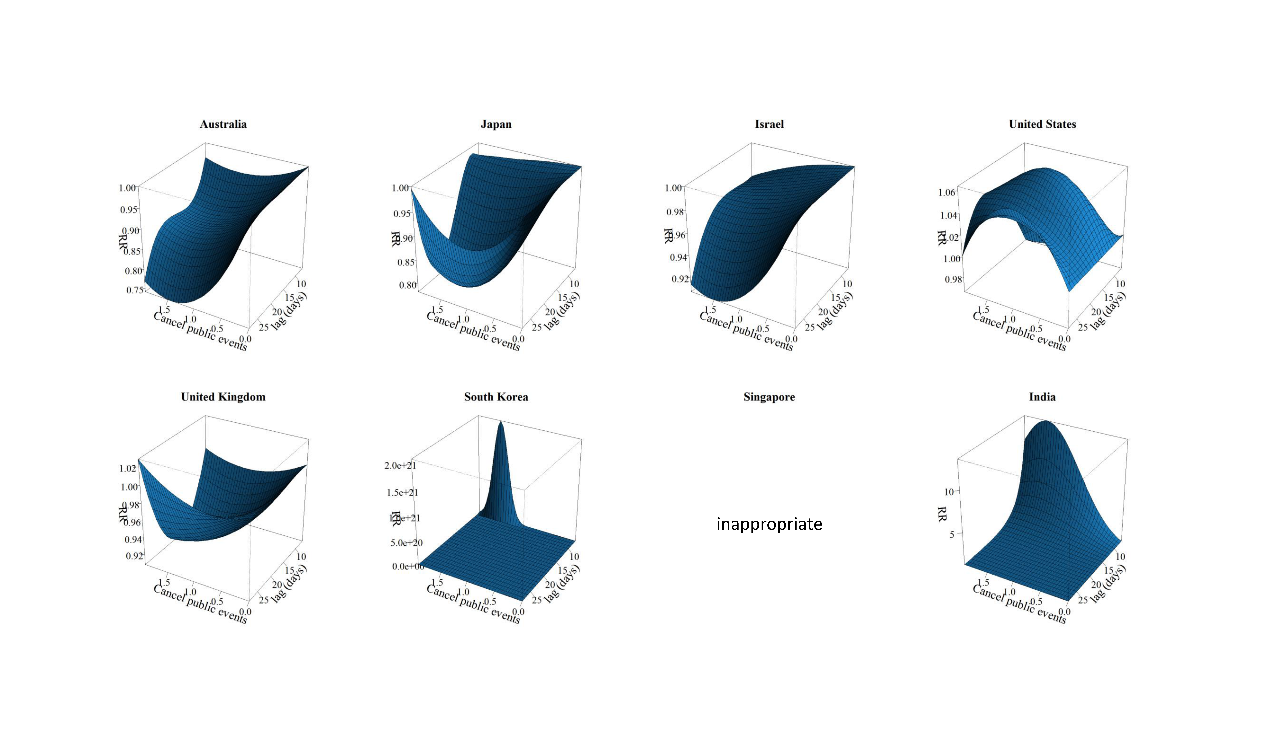
S3 Fig. The effectiveness of the restrictions on public events policy (C3).
