# Supplementary material for: Effects of vaccination and non-pharmaceutical interventions and their lag times on the COVID-19 pandemic: Comparison of eight countries
Source: PLoS Negl Trop Dis. 2022 Jan 13;16(1):e0010101. doi: 10.1371/journal.pntd.0010101 (PMC8757886; doi:10.1371/journal.pntd.0010101)
Supplement: S4 Fig — (DOCX) [file pntd.0010101.s004.docx]

S4 Fig shows that the restrictions on gatherings policy (C4) was protective for the majority of countries (RR<1), dangerous for Israel (RR>1) and the United Kingdom, and ineffective for India (RR 0.99~1.02).


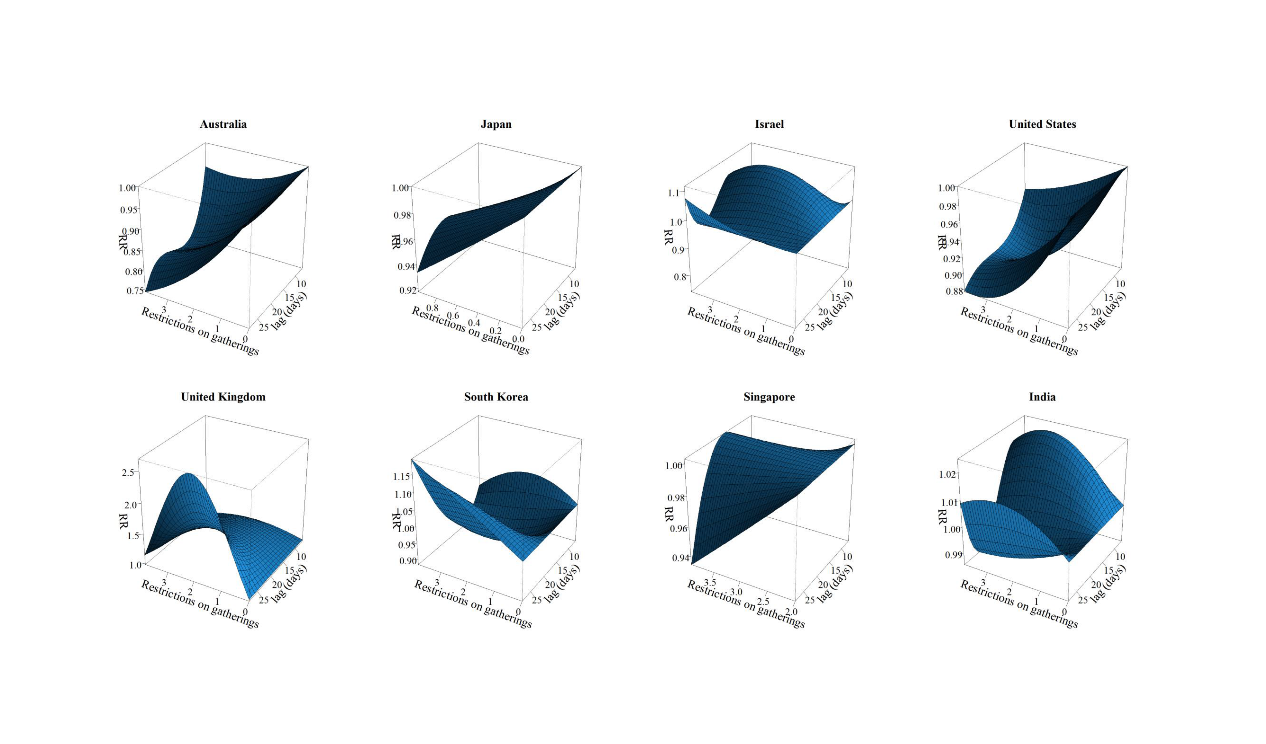
S4 Fig. The effectiveness of the restrictions on gatherings policy (C4).
