# Supplementary material for: Effects of vaccination and non-pharmaceutical interventions and their lag times on the COVID-19 pandemic: Comparison of eight countries
Source: PLoS Negl Trop Dis. 2022 Jan 13;16(1):e0010101. doi: 10.1371/journal.pntd.0010101 (PMC8757886; doi:10.1371/journal.pntd.0010101)
Supplement: S5 Fig — (DOCX) [file pntd.0010101.s005.docx]

S5 Fig shows that the closing public transport policy (C5) was dangerous for the majority of countries (RR>1) and ineffective in the United Kingdom (RR 0.98~1.02). As the C5 policy was not adopted in South Korea or Singapore, it could not be evaluated for these countries.


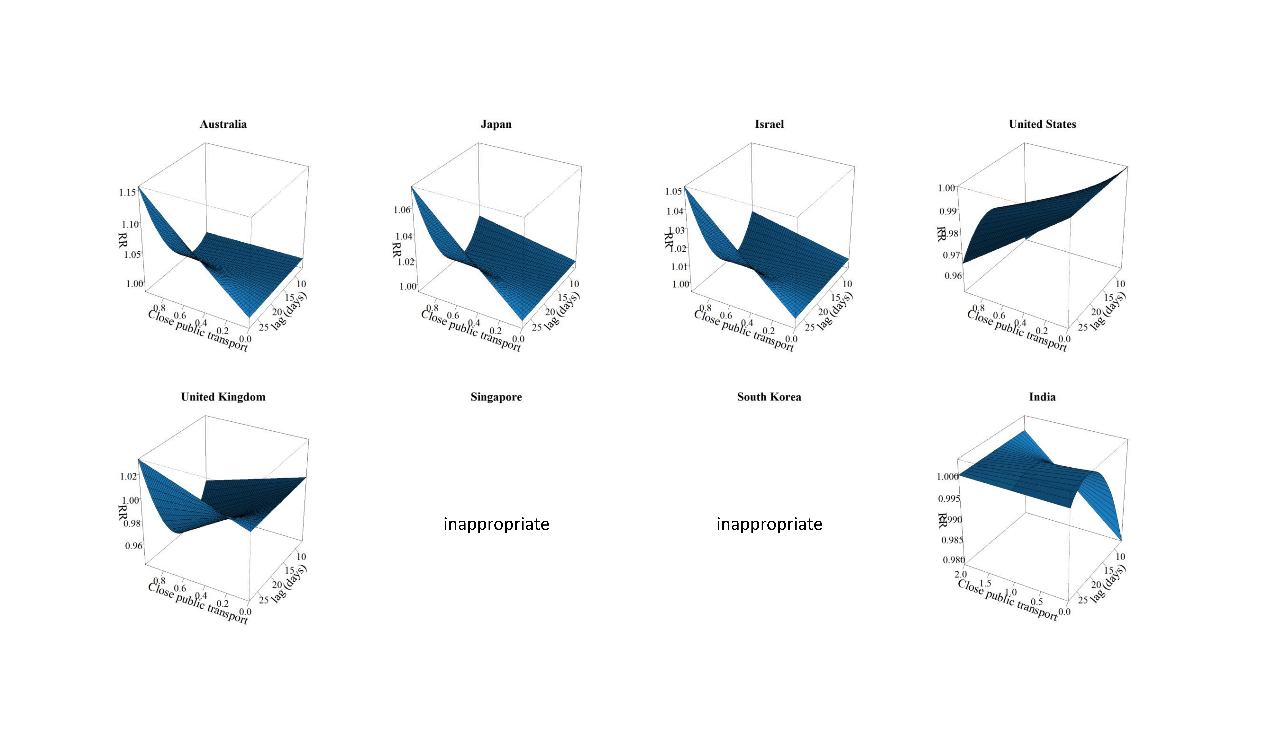
S5 Fig. The effectiveness of the closing public transport policy (C5).
