# Supplementary material for: Effects of vaccination and non-pharmaceutical interventions and their lag times on the COVID-19 pandemic: Comparison of eight countries
Source: PLoS Negl Trop Dis. 2022 Jan 13;16(1):e0010101. doi: 10.1371/journal.pntd.0010101 (PMC8757886; doi:10.1371/journal.pntd.0010101)
Supplement: S6 Fig — (DOCX) [file pntd.0010101.s006.docx]

S6 Fig shows that the staying-at-home requirements policy (C6) was protective for the majority of countries (RR<1), dangerous for South Korea (RR>1), and ineffective for India (RR 1.00~1.03).


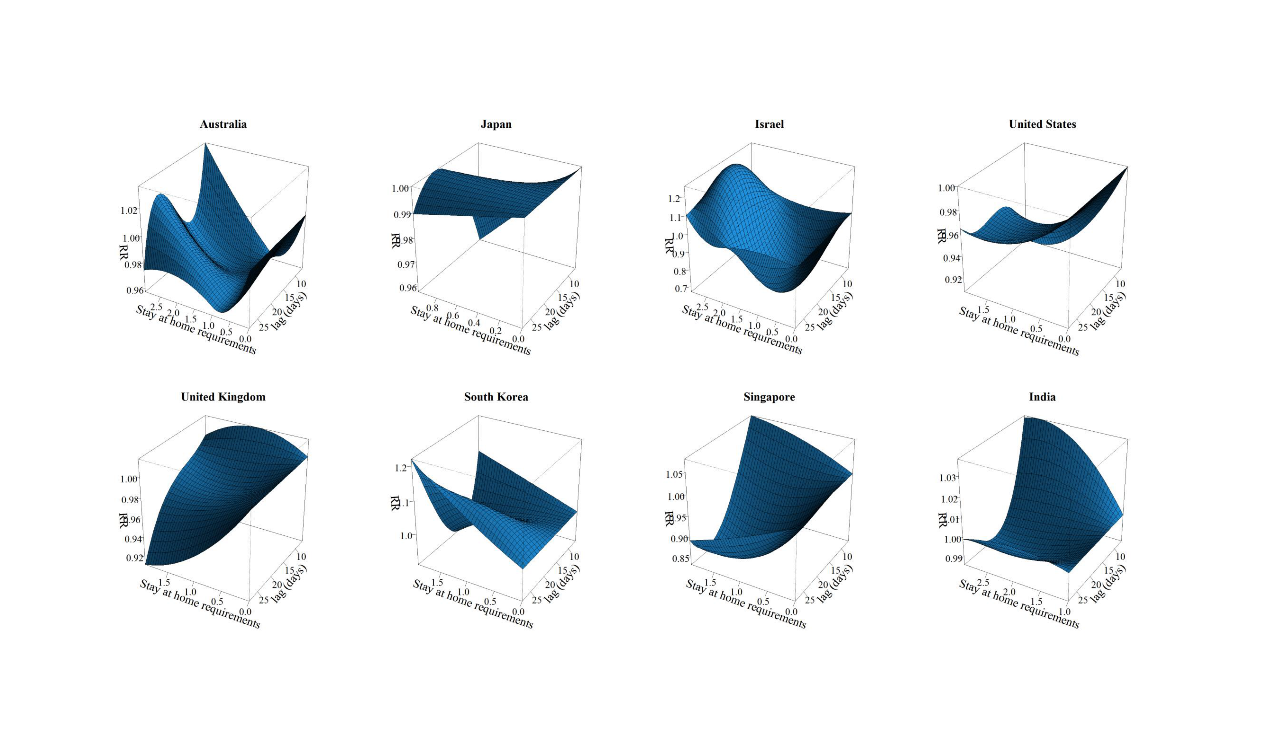
S6 Fig. The effectiveness of the staying-at-home requirements policy (C6).
