# Supplementary material for: Effects of vaccination and non-pharmaceutical interventions and their lag times on the COVID-19 pandemic: Comparison of eight countries
Source: PLoS Negl Trop Dis. 2022 Jan 13;16(1):e0010101. doi: 10.1371/journal.pntd.0010101 (PMC8757886; doi:10.1371/journal.pntd.0010101)
Supplement: S7 Fig — (DOCX) [file pntd.0010101.s007.docx]

S7 Fig shows that the restrictions on internal movement policy (C7) was protective for Israel and the United States (RR<1), dangerous for Australia and South Korea (RR>1), and ineffective for Japan, the United Kingdom and India.


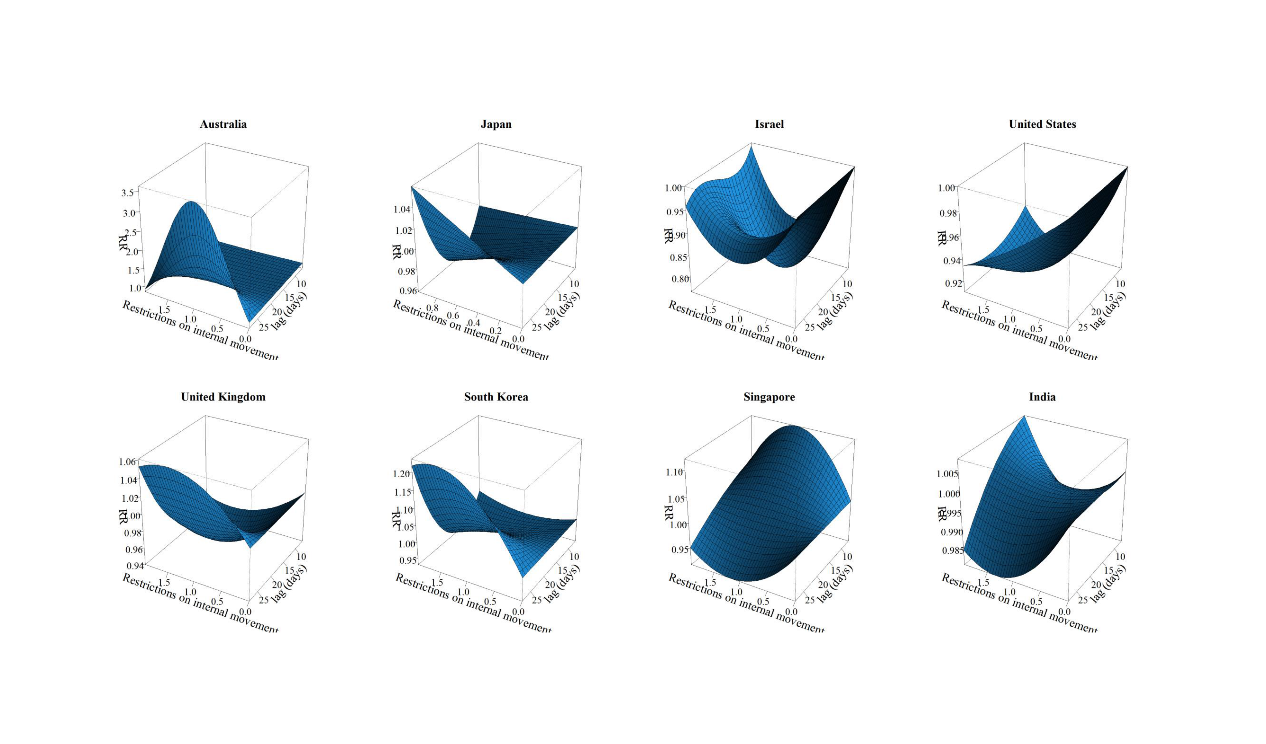
S7 Fig. The effectiveness of the restrictions on internal movement policy (C7).
