# Supplementary material for: Effects of vaccination and non-pharmaceutical interventions and their lag times on the COVID-19 pandemic: Comparison of eight countries
Source: PLoS Negl Trop Dis. 2022 Jan 13;16(1):e0010101. doi: 10.1371/journal.pntd.0010101 (PMC8757886; doi:10.1371/journal.pntd.0010101)
Supplement: S8 Fig — (DOCX) [file pntd.0010101.s008.docx]

S8 Fig shows that the international travel controls policy (C8) was protective for Australia (RR<1), dangerous for Japan, and ineffective for Israel, the United States, the United Kingdom, and South Korea (RR>1). As Singapore remained at travel control level 3 throughout, the effects could not be evaluated for this country.


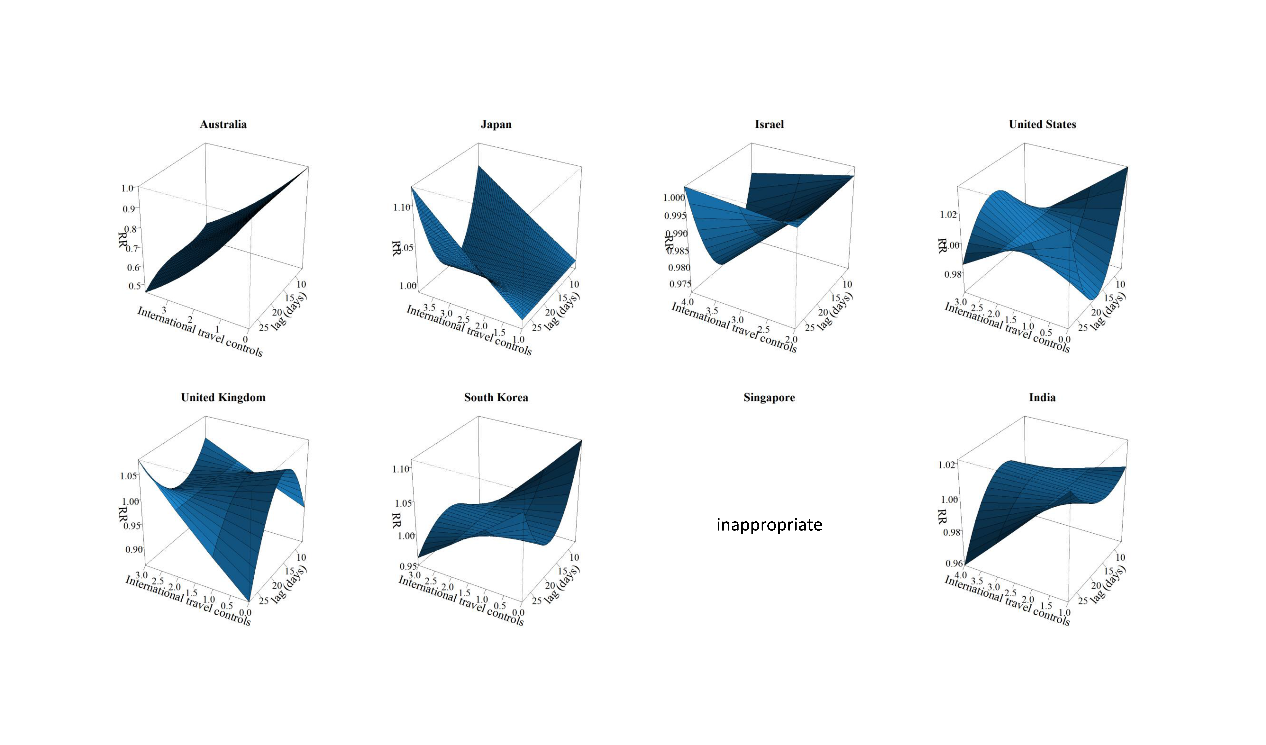


S8 Fig. The effectiveness of the international travel controls policy (C8).
