# Supplementary material for: Effects of vaccination and non-pharmaceutical interventions and their lag times on the COVID-19 pandemic: Comparison of eight countries
Source: PLoS Negl Trop Dis. 2022 Jan 13;16(1):e0010101. doi: 10.1371/journal.pntd.0010101 (PMC8757886; doi:10.1371/journal.pntd.0010101)
Supplement: S9 Fig — (DOCX) [file pntd.0010101.s009.docx]

**Australia:** After the C4 and C6 policies were canceled in June to July 2020, the effective reproductive number (Rt) increased from 0.9 to 2.0 and the daily new cases increased to a second peak of 20 per million. When the C4 and C6 policies were re-initiated, the daily new cases bottomed out at 1 per million and remained stable at this level even as the stringency index gradually declined. However, Delta variant reached a proportion over 85% in July 2021 and Australia is currently experiencing a third wave of disease even with the joint implementation of the four verified policies, with a rate of 45 daily new cases per million as of August 31, 2021.


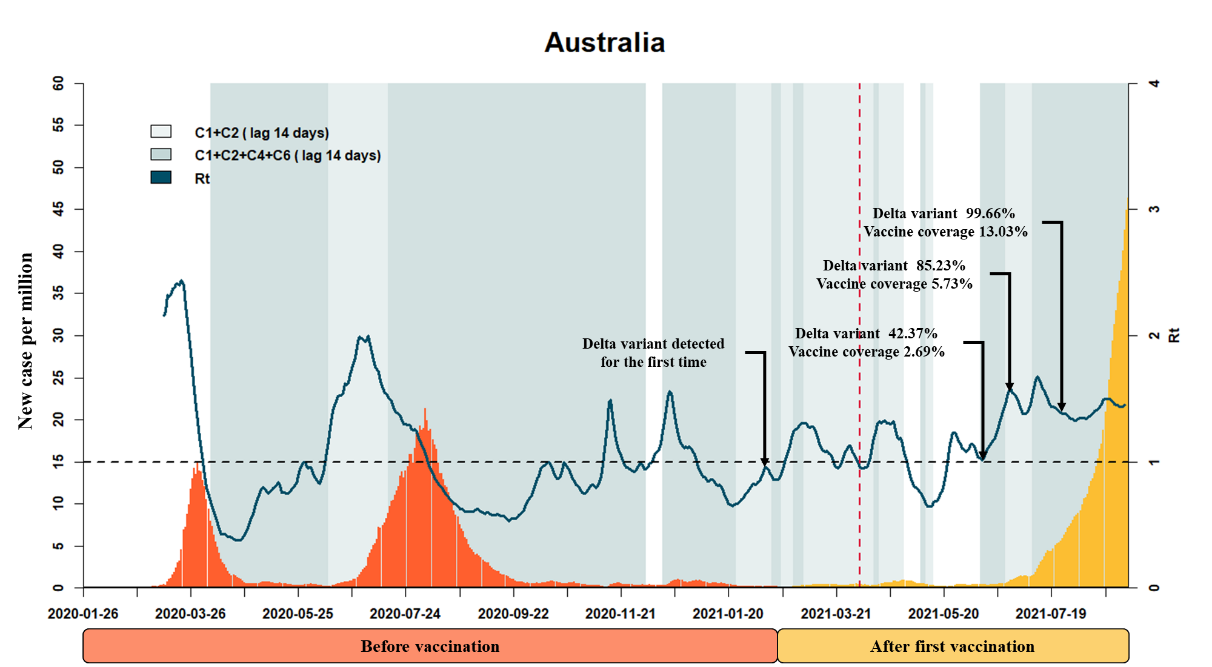


S9 Fig. Association of vaccine coverage with R_t_, new cases per million, containment and closure policies stringency index and Delta variant proportion in Australia.
