# Supplementary material for: Effects of vaccination and non-pharmaceutical interventions and their lag times on the COVID-19 pandemic: Comparison of eight countries
Source: PLoS Negl Trop Dis. 2022 Jan 13;16(1):e0010101. doi: 10.1371/journal.pntd.0010101 (PMC8757886; doi:10.1371/journal.pntd.0010101)
Supplement: S10 Fig — (DOCX) [file pntd.0010101.s010.docx]

**Israel:** After the C4 and C6 policies were canceled in June to October 2020, the daily new cases gradually increased to the first peak of 600 per million. When the four verified policies were re-implemented, this rate declined to 90 per million. After the first vaccine dose, the daily new cases increased to a second peak of 1400 per million in January 2021, even under the joint implementation of the four policies. After a 40-day lag for the onset of vaccination effect (dotted vertical line), the daily new cases dropped and maintained a low level despite removal of the C4 and C6 policies. However, the Delta variant proportion exceeded 68% in June 2021, and Israel is currently experiencing a third wave of disease, with a rate of 1000 daily new cases per million as of 31 August, 2021.


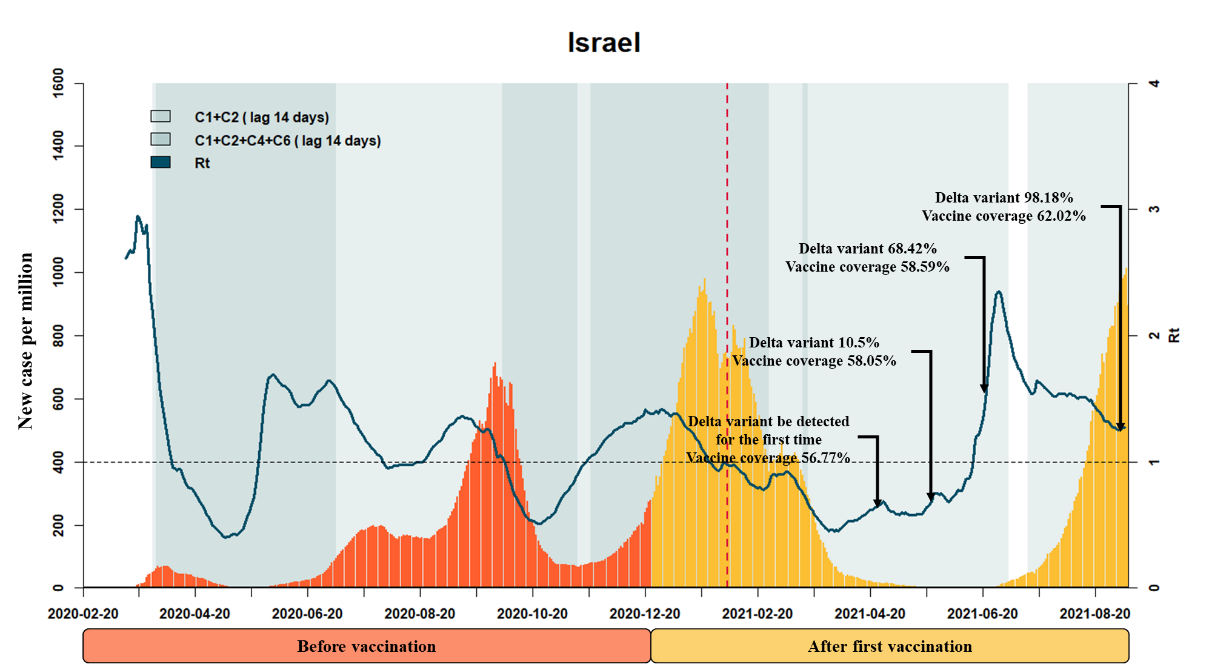


S10 Fig. Association of vaccine coverage with R_t_, new cases per million, containment and closure policies stringency index and Delta variant proportion in Israel.
