# Supplementary material for: Effects of vaccination and non-pharmaceutical interventions and their lag times on the COVID-19 pandemic: Comparison of eight countries
Source: PLoS Negl Trop Dis. 2022 Jan 13;16(1):e0010101. doi: 10.1371/journal.pntd.0010101 (PMC8757886; doi:10.1371/journal.pntd.0010101)
Supplement: S11 Fig — (DOCX) [file pntd.0010101.s011.docx]

**Singapore:** After the joint implementation of the four verified policies in April 23, 2020, the rate of daily new cases in Singapore gradually bottomed out and maintained a low level beginning in October 2020. The Delta variant proportion exceeded 99% in July 2021, and Singapore is currently experiencing a small second wave, with 30 new daily cases per million as of August 2021.


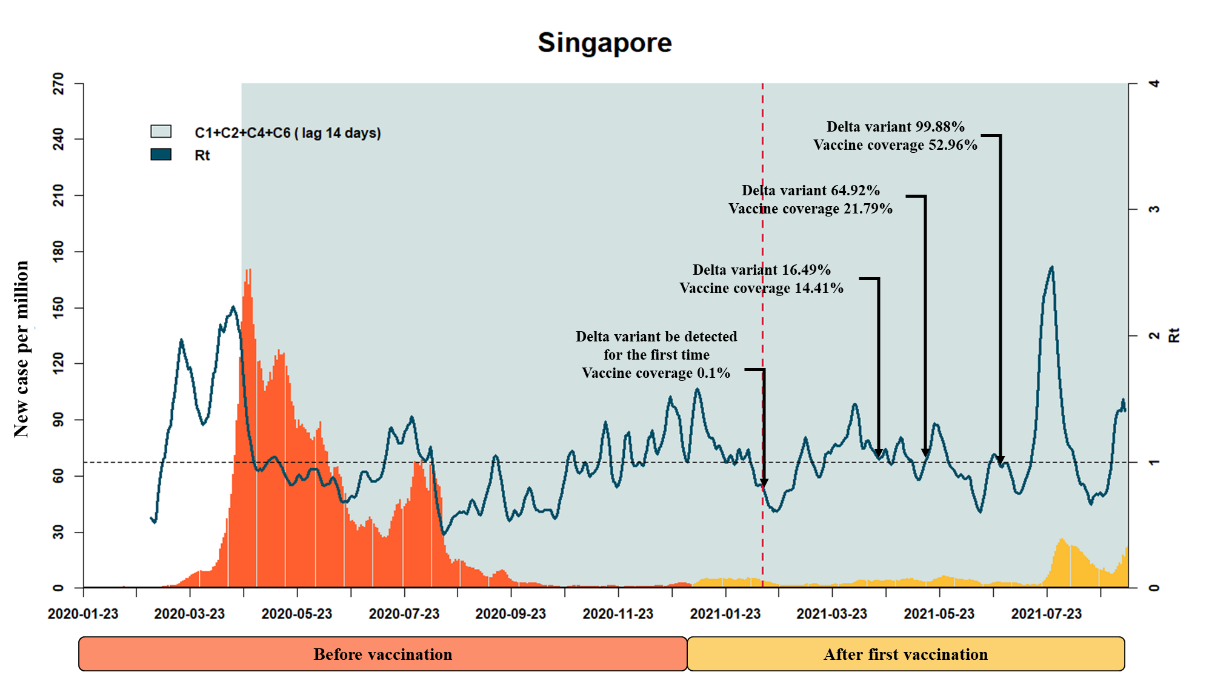


S11 Fig. Association of vaccine coverage with R_t_, new cases per million, containment and closure policies stringency index and Delta variant proportion in Singapore.
