# Supplementary material for: Effects of vaccination and non-pharmaceutical interventions and their lag times on the COVID-19 pandemic: Comparison of eight countries
Source: PLoS Negl Trop Dis. 2022 Jan 13;16(1):e0010101. doi: 10.1371/journal.pntd.0010101 (PMC8757886; doi:10.1371/journal.pntd.0010101)
Supplement: S12 Fig — (DOCX) [file pntd.0010101.s012.docx]

**United Kingdom:** The United Kingdom experienced a first wave of disease from March to June 2020, controlled the disease until October 2020, then experienced a second peak of 300 daily new cases per million in November 2020. After the first vaccine dose, the rate increased to a third peak of 800 per million. After a 40-day lag for the onset of vaccination effect (dotted vertical line), Rt<1 and the daily new cases gradually dropped to a low level that was maintained even when the C4 and C6 policies were canceled. However, the Delta variant proportion exceeded 73% in June 2021, and the United Kingdom is currently experiencing a fourth wave of disease. The UK nearly had joint implementation of the four verified policies before the first three waves, but had C1 and C2 policies since May 2021.


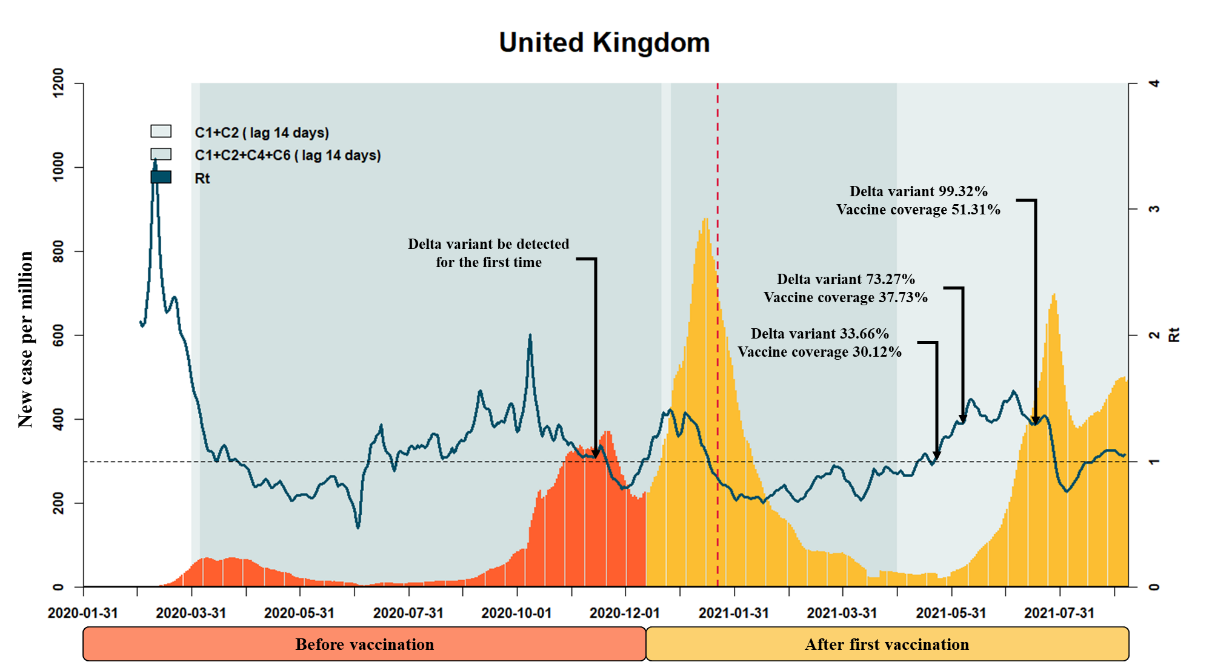


S12 Fig. Association of vaccine coverage with R_t_, new cases per million, containment and closure policies stringency index and Delta variant proportion in the United Kingdom.
