# Supplementary material for: Effects of vaccination and non-pharmaceutical interventions and their lag times on the COVID-19 pandemic: Comparison of eight countries
Source: PLoS Negl Trop Dis. 2022 Jan 13;16(1):e0010101. doi: 10.1371/journal.pntd.0010101 (PMC8757886; doi:10.1371/journal.pntd.0010101)
Supplement: S13 Fig — (DOCX) [file pntd.0010101.s013.docx]

**India:** India had joint implementation of the four verified policies throughout most of the study period. The first peak of 70 daily new cases per million occurred in September 2020. After March 2021, with the rapid increase of Delta variant and a slow vaccination process, the daily new cases rocketed to a second peak of 280 per million in April 2021. This rate then declined to a second minimum in July 2021.

**
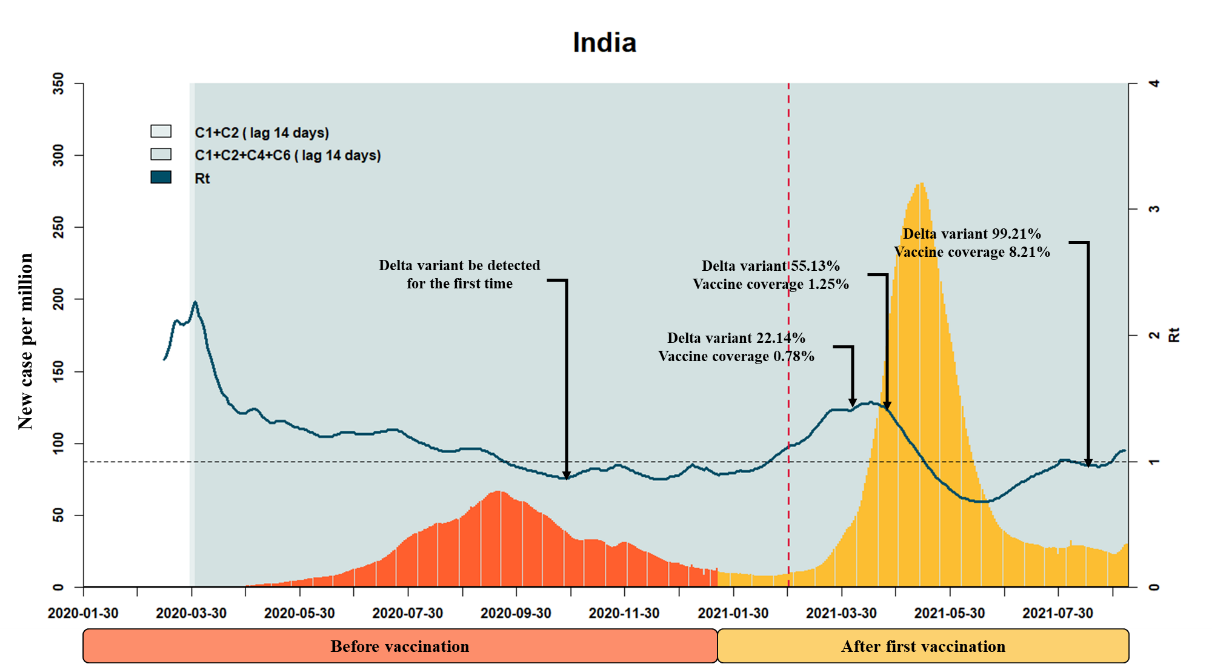
**

S13 Fig. Association of vaccine coverage with R_t_, new cases per million, containment and closure policies stringency index and Delta variant proportion in India.
