# Supplementary material for: Effects of vaccination and non-pharmaceutical interventions and their lag times on the COVID-19 pandemic: Comparison of eight countries
Source: PLoS Negl Trop Dis. 2022 Jan 13;16(1):e0010101. doi: 10.1371/journal.pntd.0010101 (PMC8757886; doi:10.1371/journal.pntd.0010101)
Supplement: S14 Fig — (DOCX) [file pntd.0010101.s014.docx]

**United States:** The United States also had joint implementation of the four verified policies throughout most of the study period. The daily new cases gradually increased to a peak of 650 per million in December 2020. After the first dose of the vaccine, the daily new cases continuously increased to a peak of 750 per million in January 2021. After a 40-day lag time for the onset of vaccination effect (dotted vertical line), the daily new cases gradually dropped to a minimum of 50 per million in June 2021. However, with the proportion of Delta variant over 50%, the United States is currently experiencing a second wave of disease, with 450 daily new cases per million as of August 31, 2021.


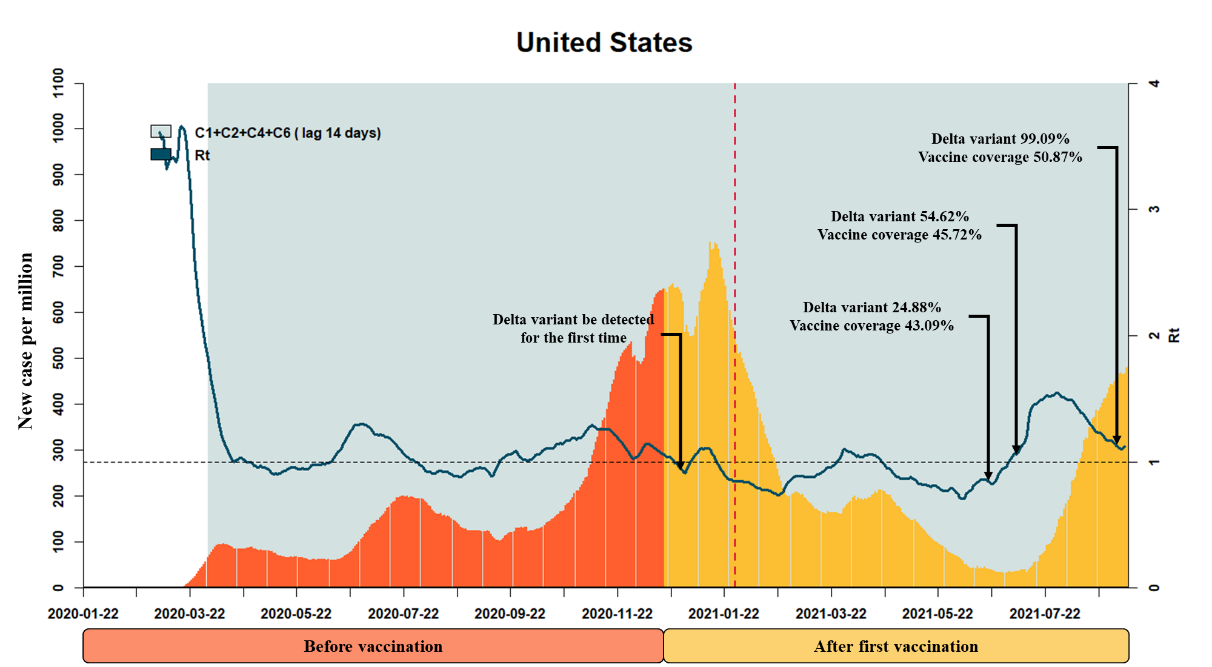


S14 Fig. Association of vaccine coverage with R_t_, new cases per million, containment and closure policies stringency index and Delta variant proportion in the United States.
