# Supplementary material for: Effects of vaccination and non-pharmaceutical interventions and their lag times on the COVID-19 pandemic: Comparison of eight countries
Source: PLoS Negl Trop Dis. 2022 Jan 13;16(1):e0010101. doi: 10.1371/journal.pntd.0010101 (PMC8757886; doi:10.1371/journal.pntd.0010101)
Supplement: S15 Fig — (DOCX) [file pntd.0010101.s015.docx]

**South Korea:** After the C4 and C6 policies were canceled in May to June 2020, the Rt fluctuated but the rate of daily new cases remained stable. Starting in November 2020, the daily new cases increased to a first peak of 20 per million. After the first dose of vaccine, the rate declined after a 40-day lag for the onset of vaccination effect, and remained stable for some time. The Delta variant proportion exceeded 50% in June 2021, and South Korea is currently experiencing a third wave, with 35 daily new cases per million as of August 31, 2021.

**
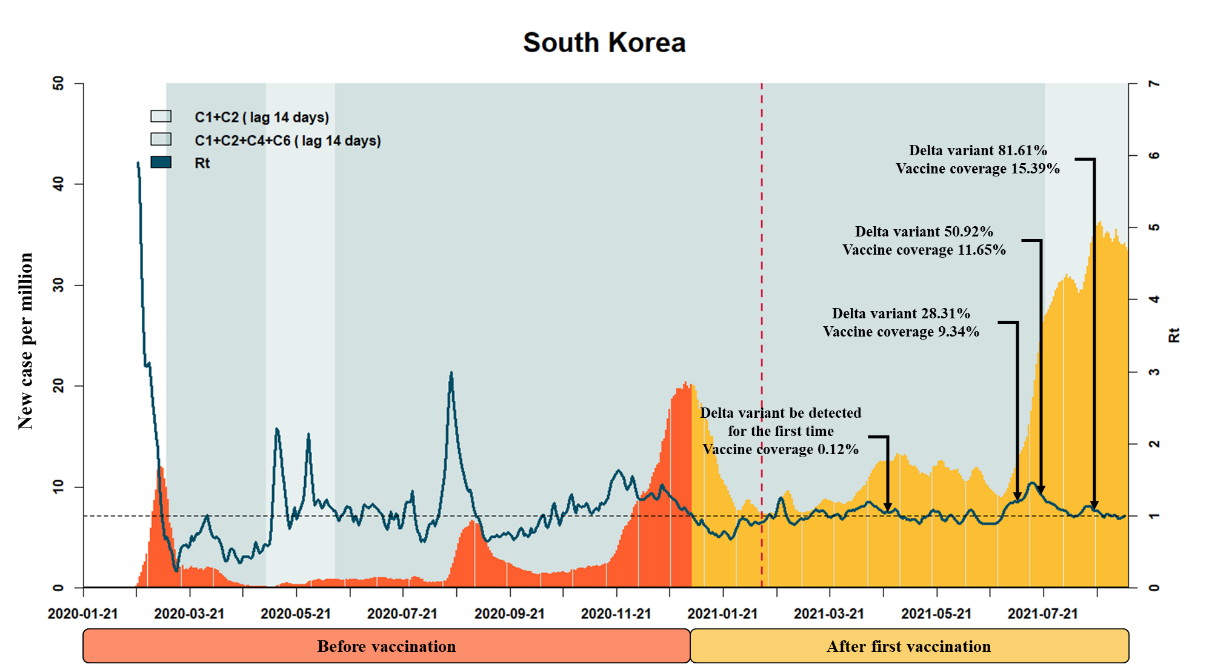
**

S15 Fig. Association of vaccine coverage with R_t_, new cases per million, containment and closure policies stringency index and Delta variant proportion in South Korea.
