# Supplementary material for: Effects of vaccination and non-pharmaceutical interventions and their lag times on the COVID-19 pandemic: Comparison of eight countries
Source: PLoS Negl Trop Dis. 2022 Jan 13;16(1):e0010101. doi: 10.1371/journal.pntd.0010101 (PMC8757886; doi:10.1371/journal.pntd.0010101)
Supplement: S16 Fig — (DOCX) [file pntd.0010101.s016.docx]

**Japan:** Japan adopted a relatively low-stringency index of containment and closure policies during the early phase of the pandemic, and the daily new cases increased in the two periods when there was no policy implemented. In January 2021, the daily new cases reached a first peak of 50 per million; the rate declined thereafter with the implementation of the C4 and C6 policies. After the first dose of vaccine, the daily new cases first increased and then after a 40-day lag for the onset of vaccination effect, began to decrease. Starting in July 2021, the daily new cases soared to a third peak of 100 per million with the increasing proportion of Delta variant.


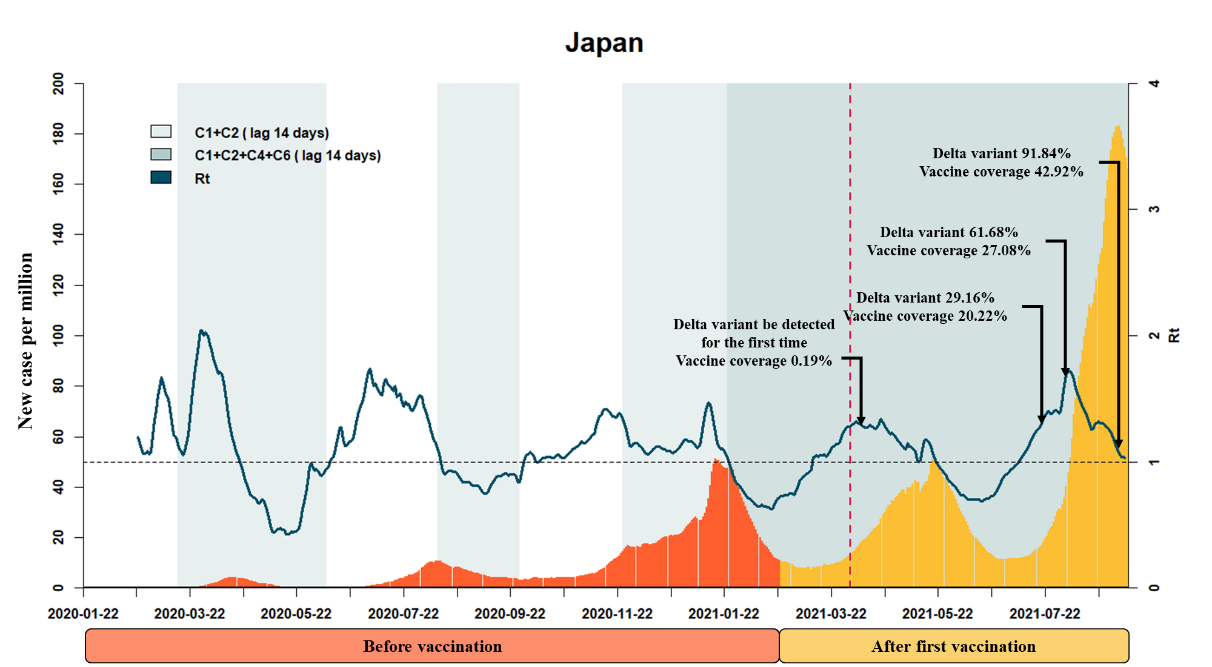


S16 Fig. Association of vaccine coverage with R_t_, new cases per million, containment and closure policies stringency index and Delta variant proportion in Japan.
