# Supplementary material for: Effects of vaccination and non-pharmaceutical interventions and their lag times on the COVID-19 pandemic: Comparison of eight countries
Source: PLoS Negl Trop Dis. 2022 Jan 13;16(1):e0010101. doi: 10.1371/journal.pntd.0010101 (PMC8757886; doi:10.1371/journal.pntd.0010101)
Supplement: S17 Fig — (DOCX) [file pntd.0010101.s017.docx]

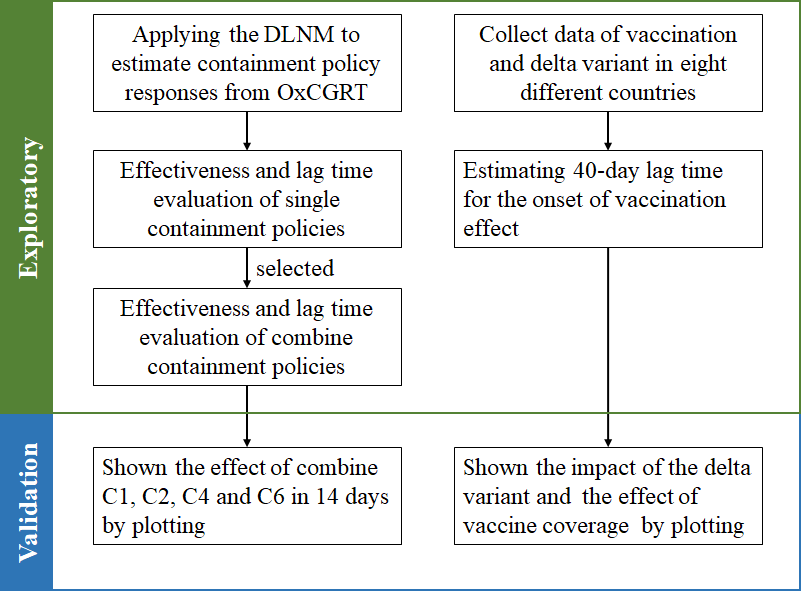


S17 Fig. Flow chart. OxCGRT, Oxford COVID-19 Government Response Tracker; C1, school closure; C2, workplace closure; C4, restrictions on mass gatherings; C6, stay-at-home requirements.
